# Supplementary figures and images for: Short heat shock factor A2 regulates heat resistance and growth balance in Arabidopsis
Source: eLife. 2025 Nov 3;13:RP99937. doi: 10.7554/eLife.99937 (PMC12582567; doi:10.7554/eLife.99937)

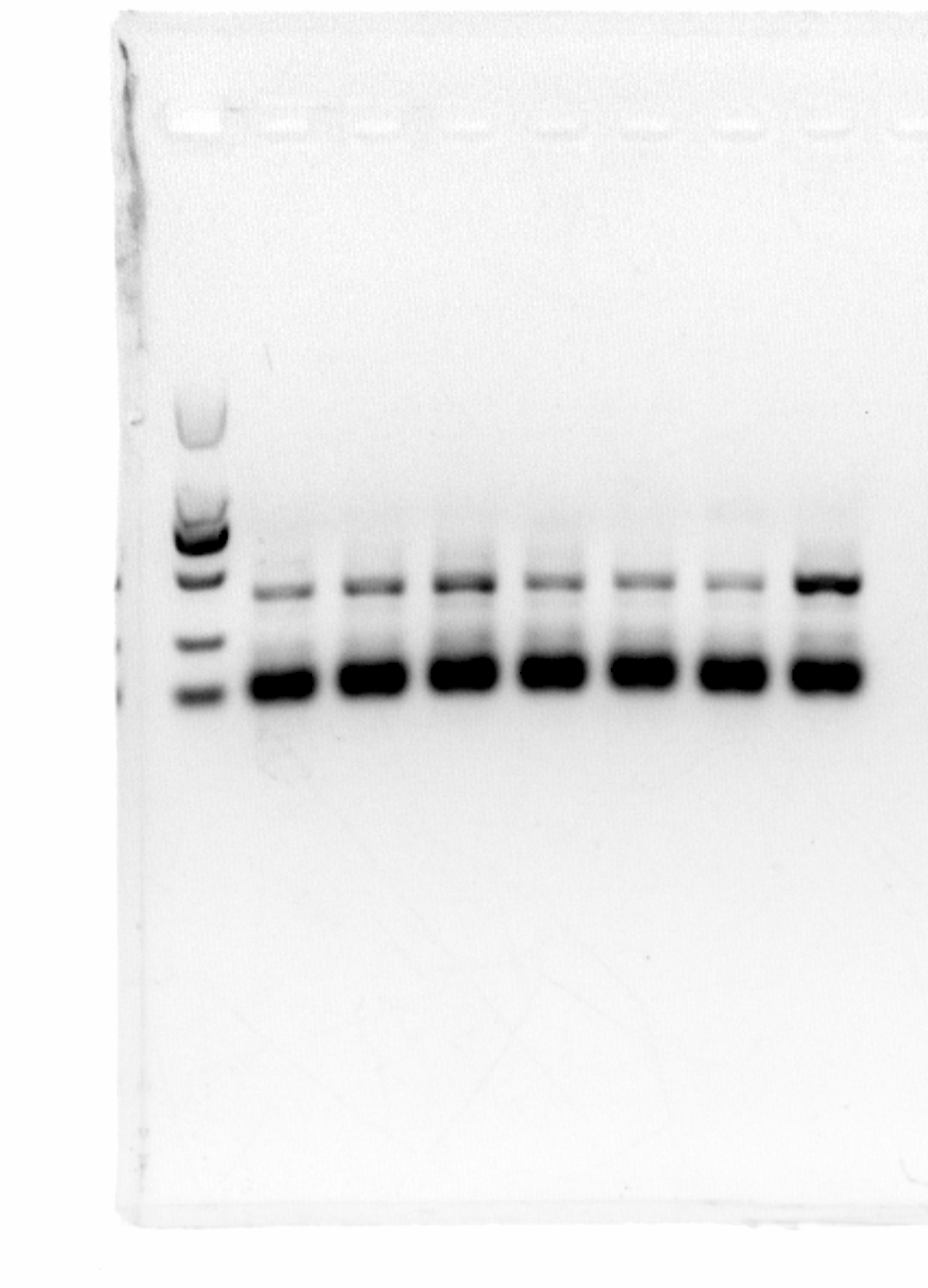

Supplement: Figure 1—source data 2. [file elife-99937-fig1-data2.zip › LLY-A2-RT-PCR.tif]

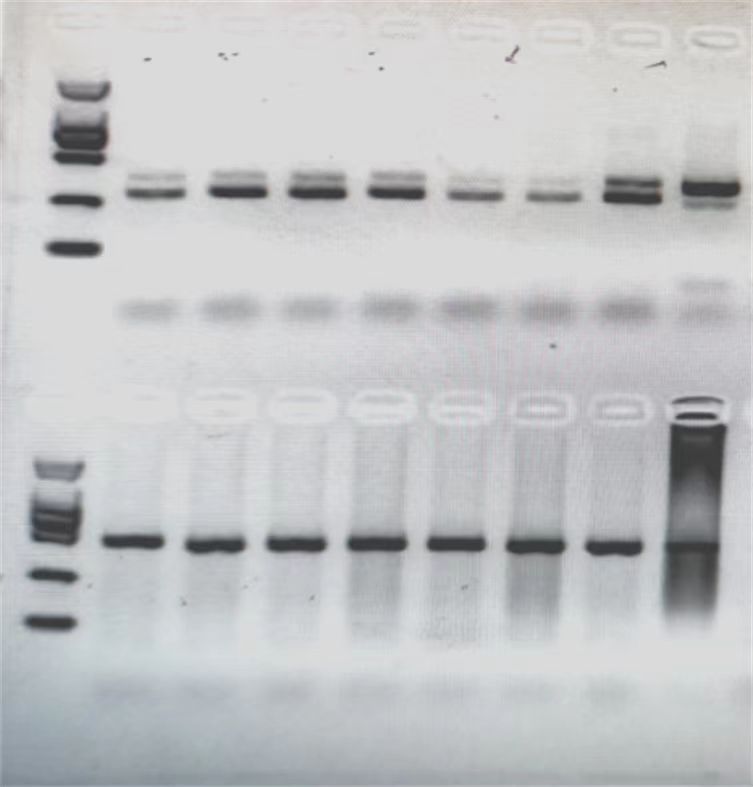

Supplement: Figure 1—source data 2. [file elife-99937-fig1-data2.zip › LLY-SA2-YLS8-RT-PCR.jpg]

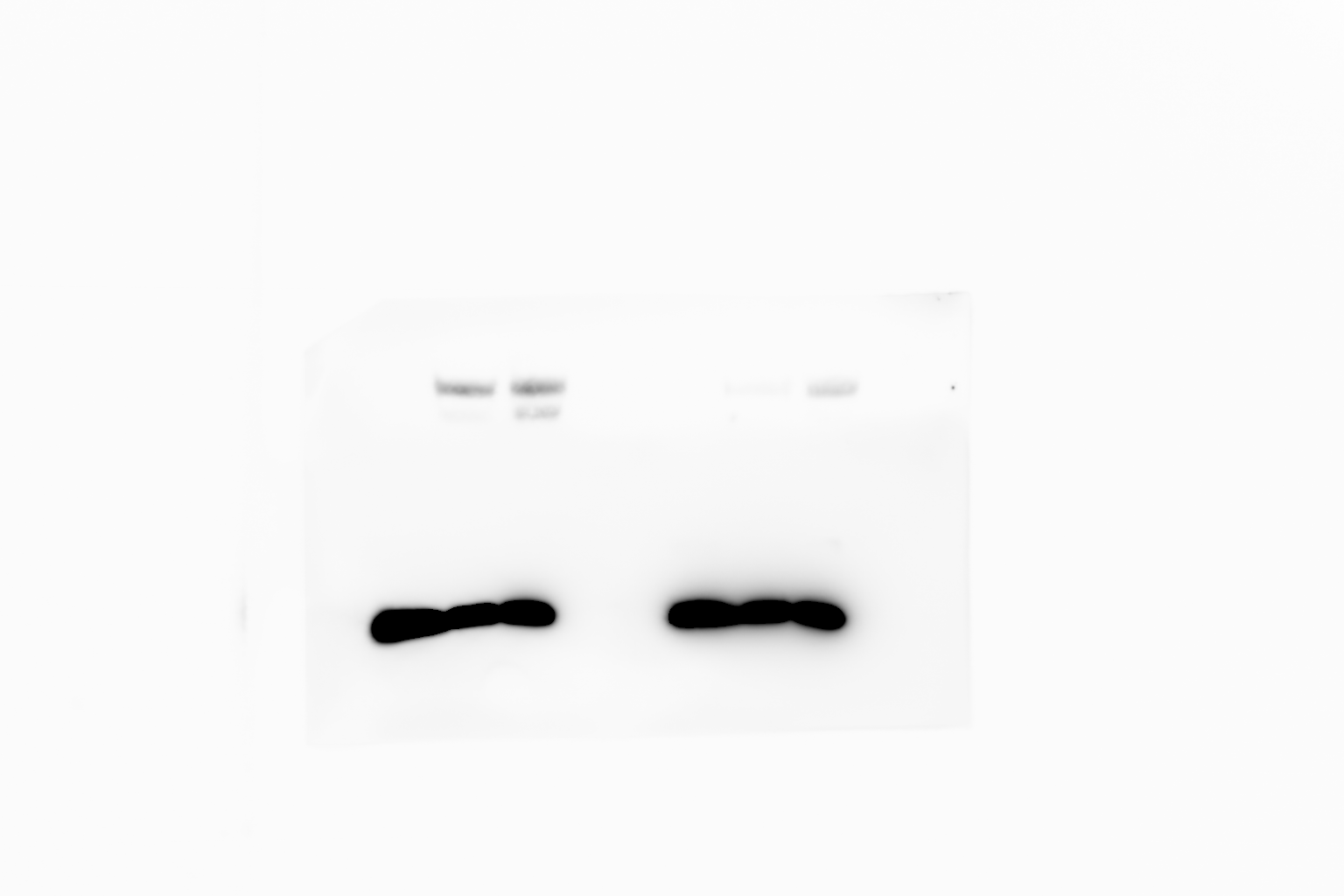

Supplement: Figure 3—source data 2. [file elife-99937-fig3-data2.zip › Figure 3C- source data-EMSA1.tif]

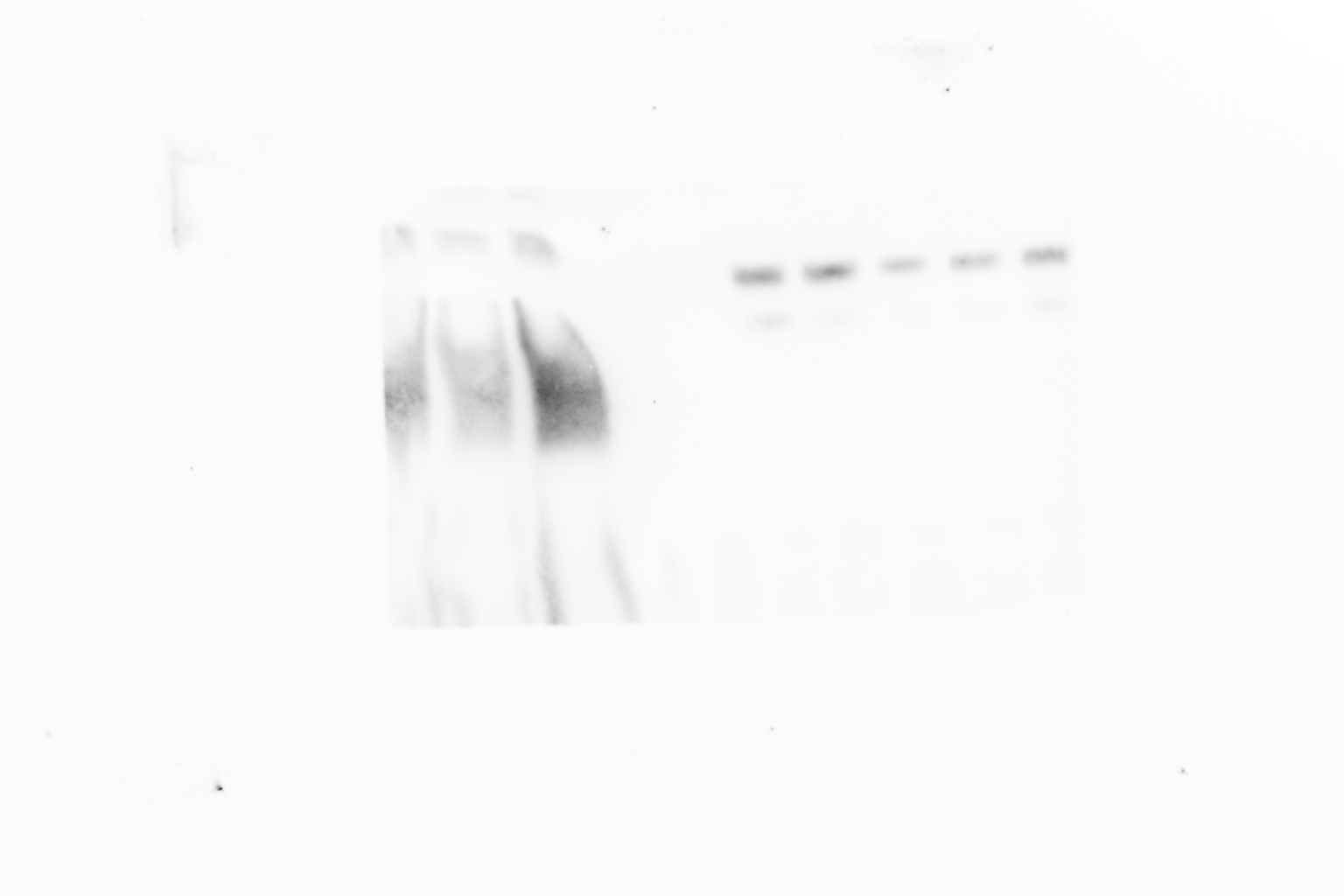

Supplement: Figure 3—source data 2. [file elife-99937-fig3-data2.zip › Figure 3C- source data-EMSA2.tif]

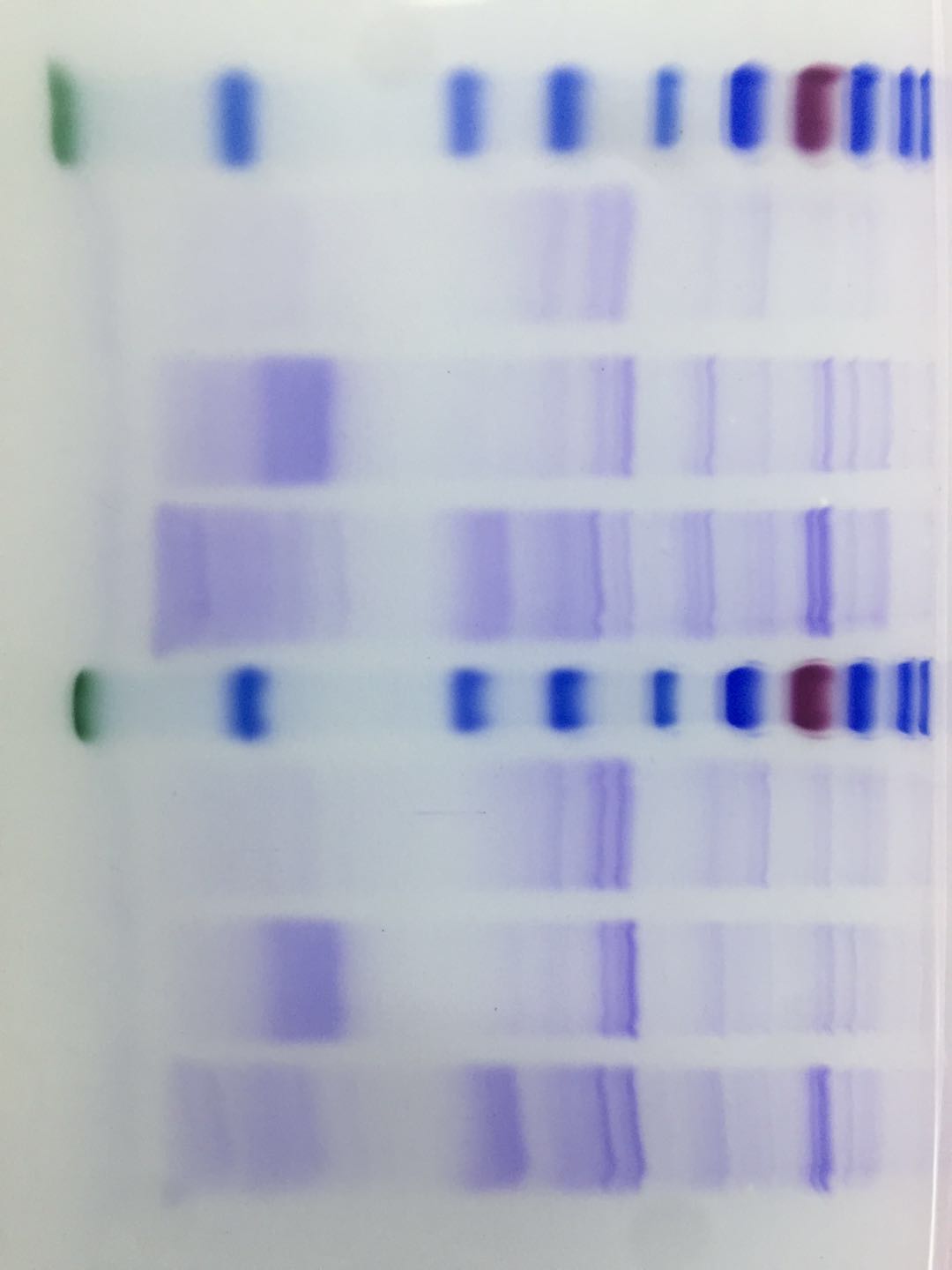

Supplement: Figure 3—source data 2. [file elife-99937-fig3-data2.zip › Figure 3D- source data-CBB.jpg]

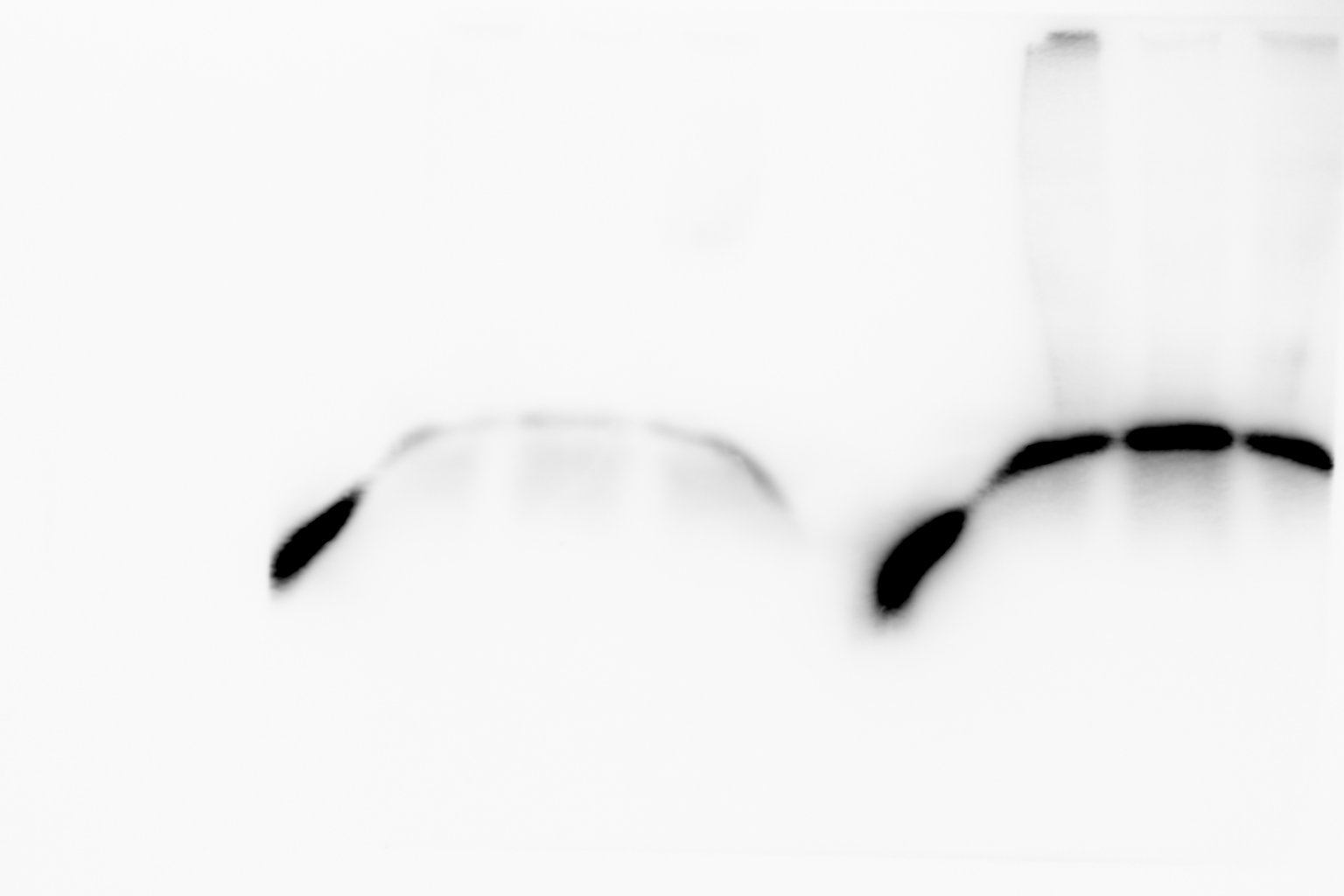

Supplement: Figure 3—source data 2. [file elife-99937-fig3-data2.zip › Figure 3D- source data-EMSA3.tif]

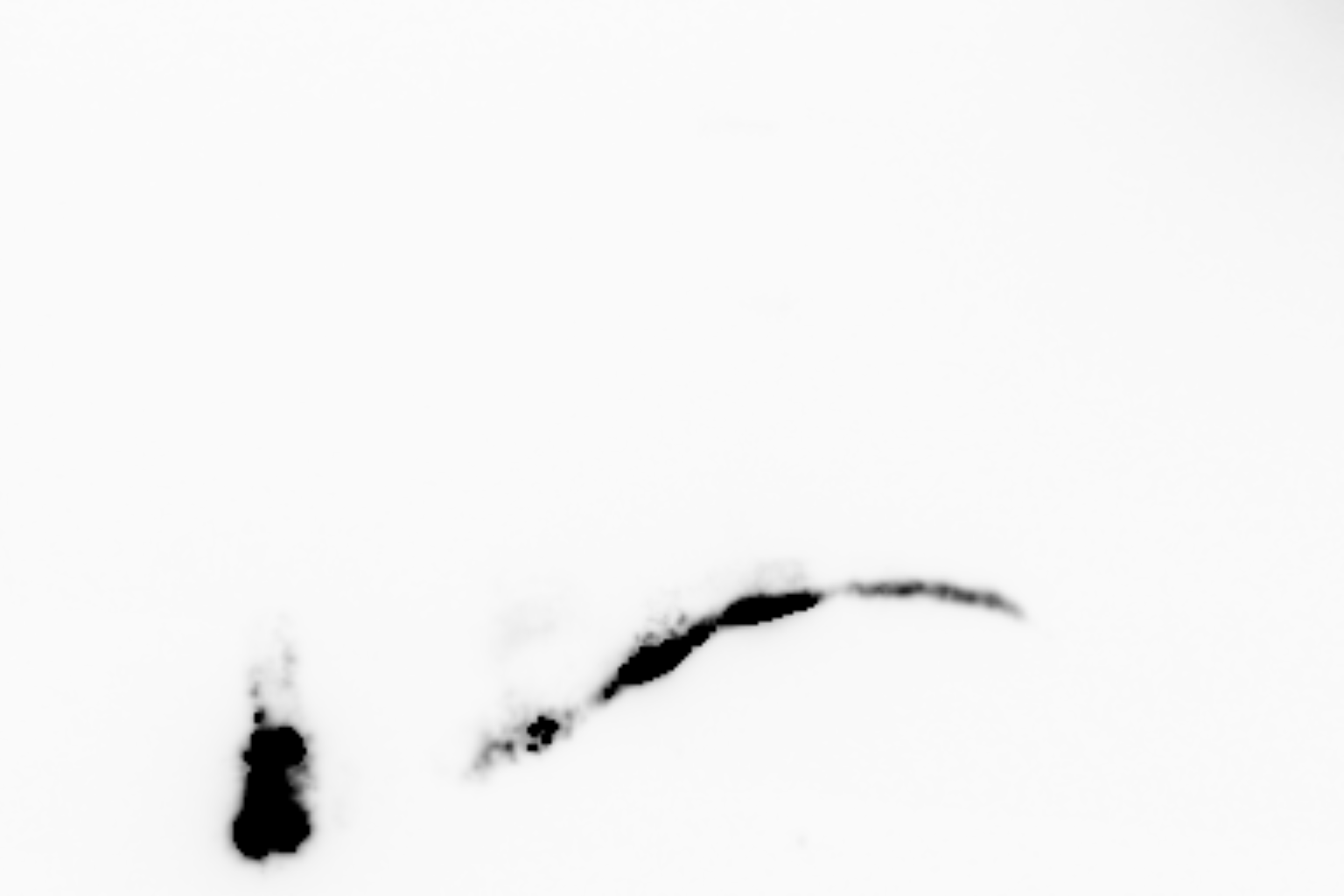

Supplement: Figure 3—source data 2. [file elife-99937-fig3-data2.zip › Figure 3D- source data-EMSA4.tif]

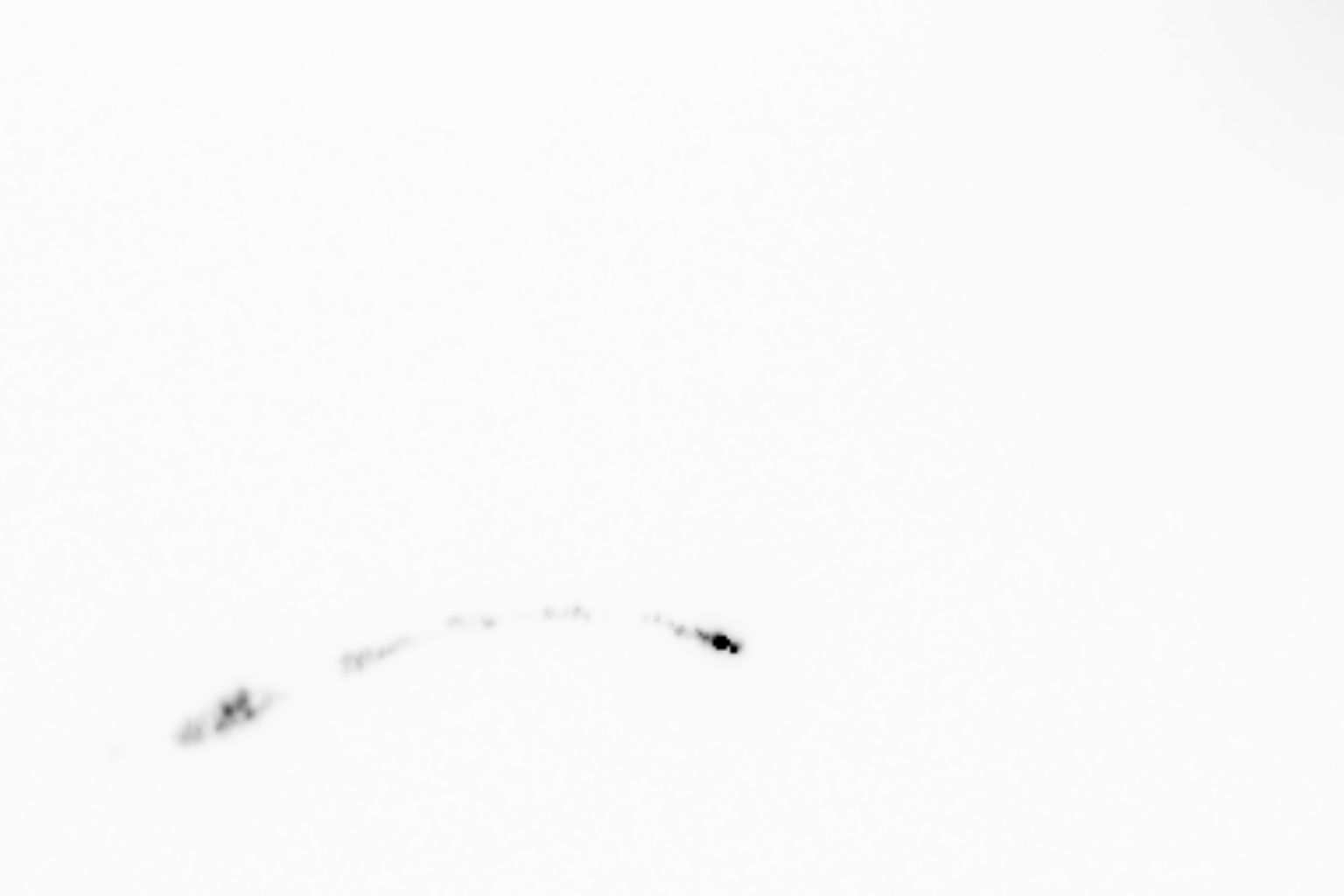

Supplement: Figure 3—source data 2. [file elife-99937-fig3-data2.zip › Figure 3D- source data-EMSA5.tif]

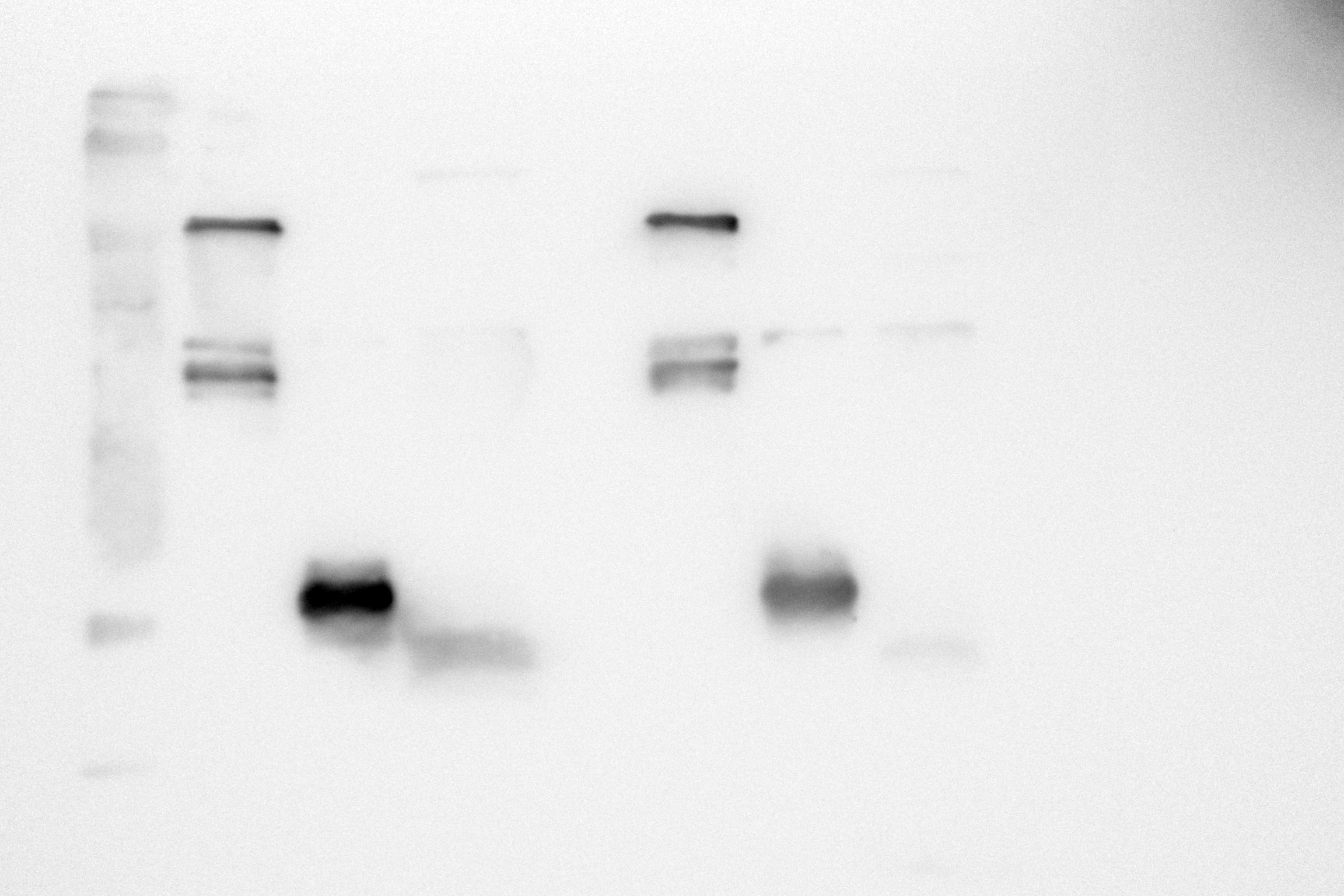

Supplement: Figure 3—source data 2. [file elife-99937-fig3-data2.zip › Figure 3D- source data-WB.tif]

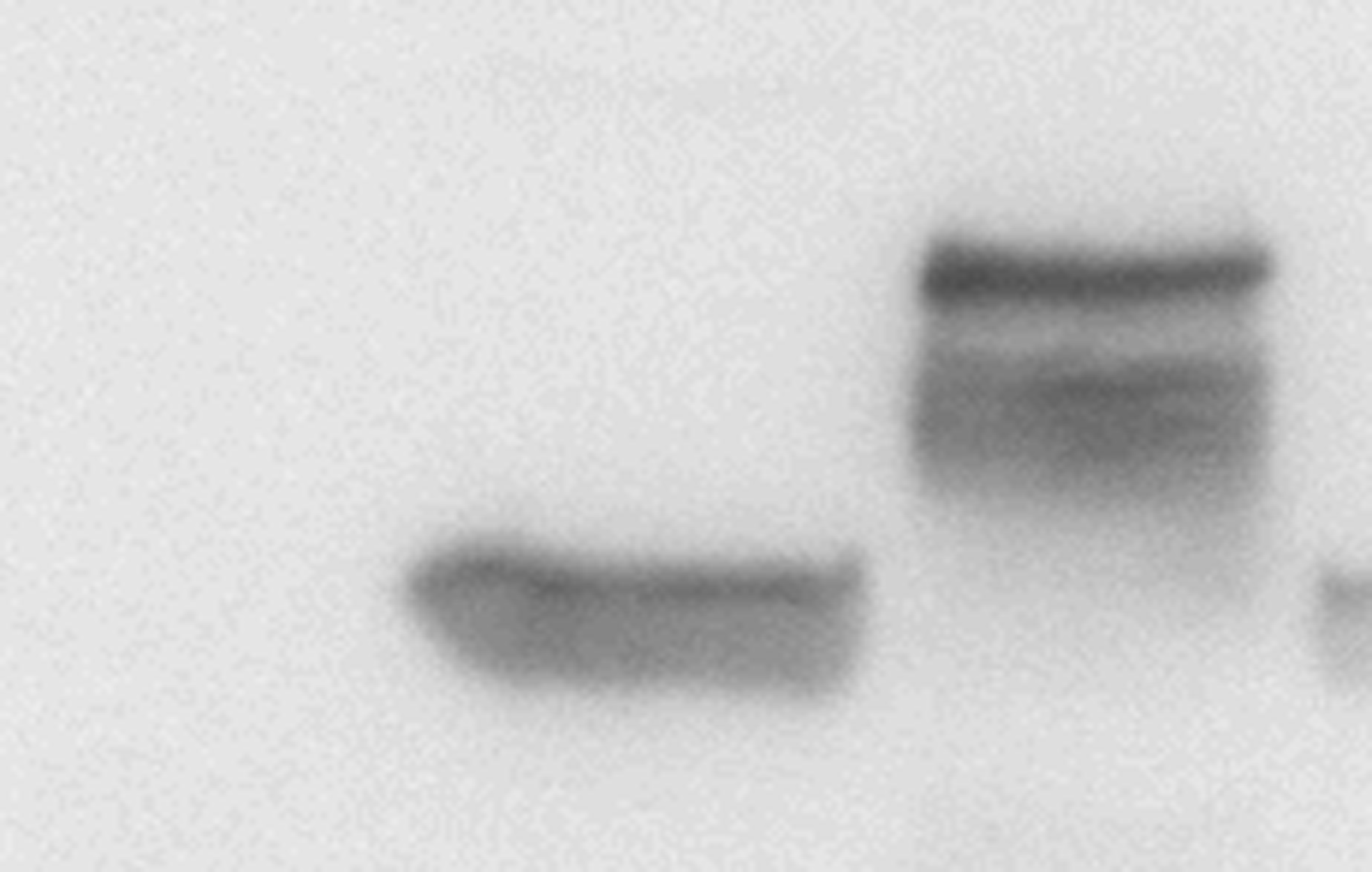

Supplement: Figure 3—figure supplement 1—source data 2. [file elife-99937-fig3-figsupp1-data2.zip › Figure 3- figure supplement 1 source data -WB.tif]

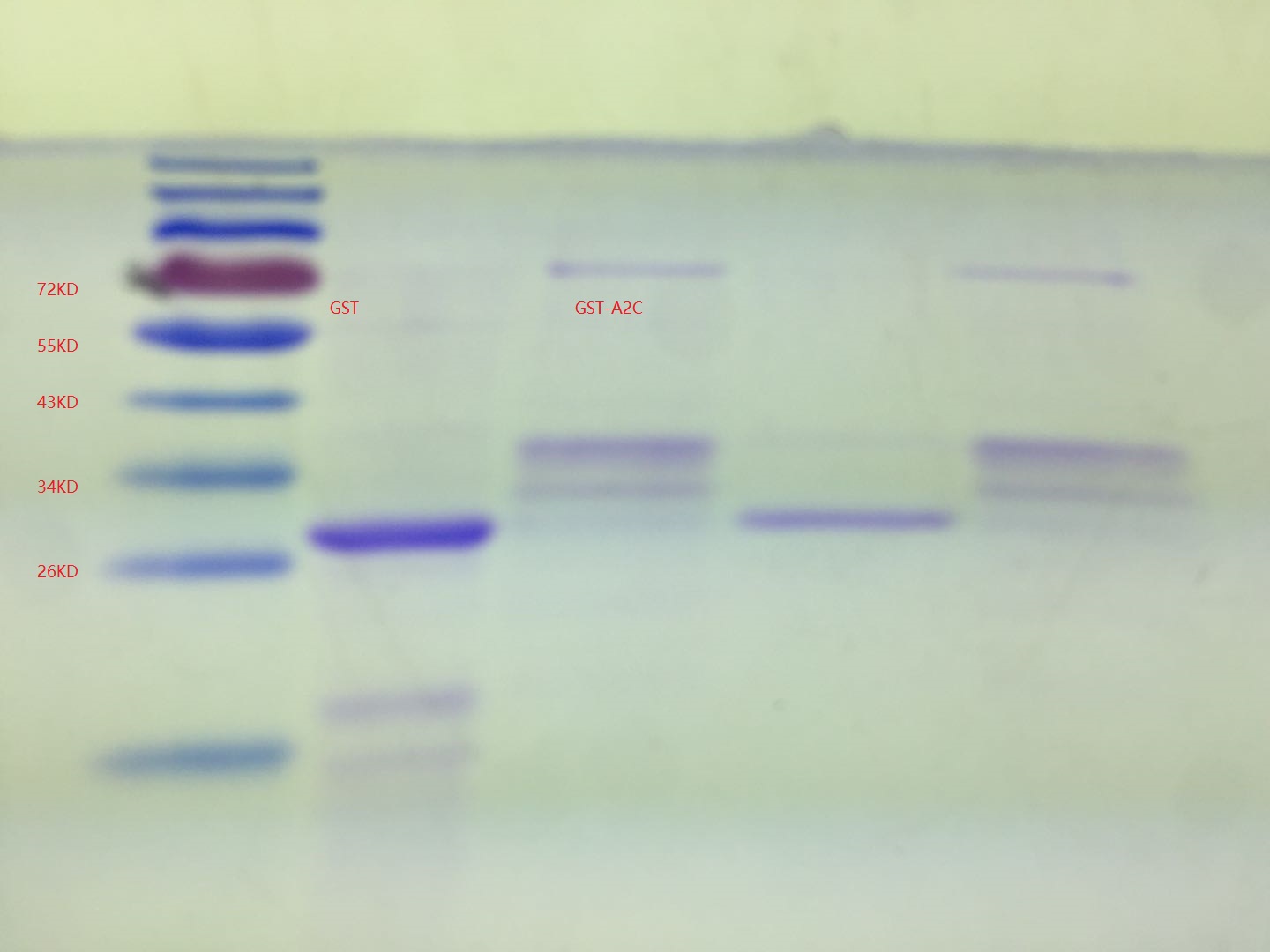

Supplement: Figure 3—figure supplement 1—source data 2. [file elife-99937-fig3-figsupp1-data2.zip › Figure 3- figure supplement 1 source data-CBB.jpg]

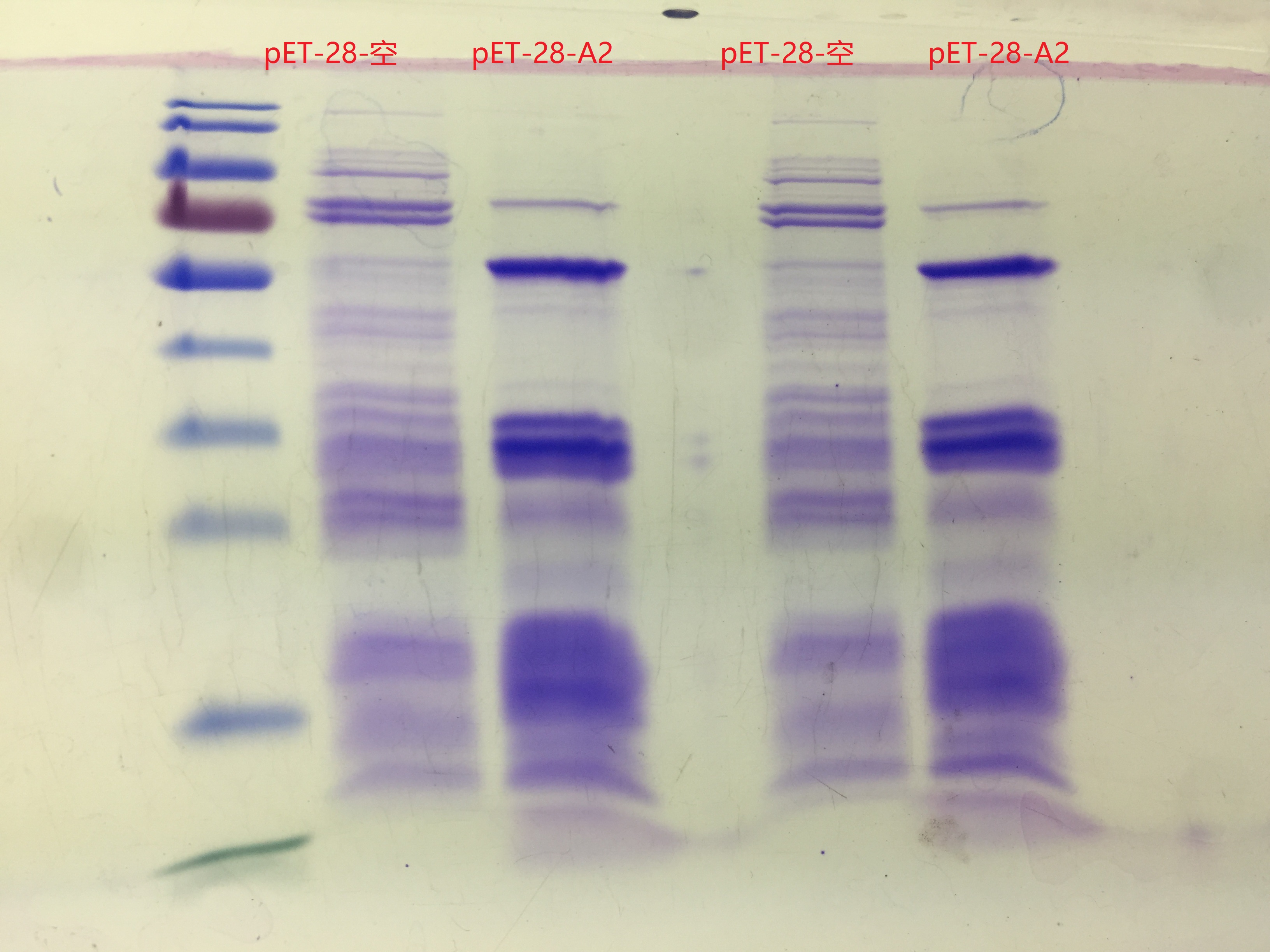

Supplement: Figure 3—figure supplement 2—source data 2. [file elife-99937-fig3-figsupp2-data2.zip › Figure 3- figure supplement 2 source data-CBB.jpg]

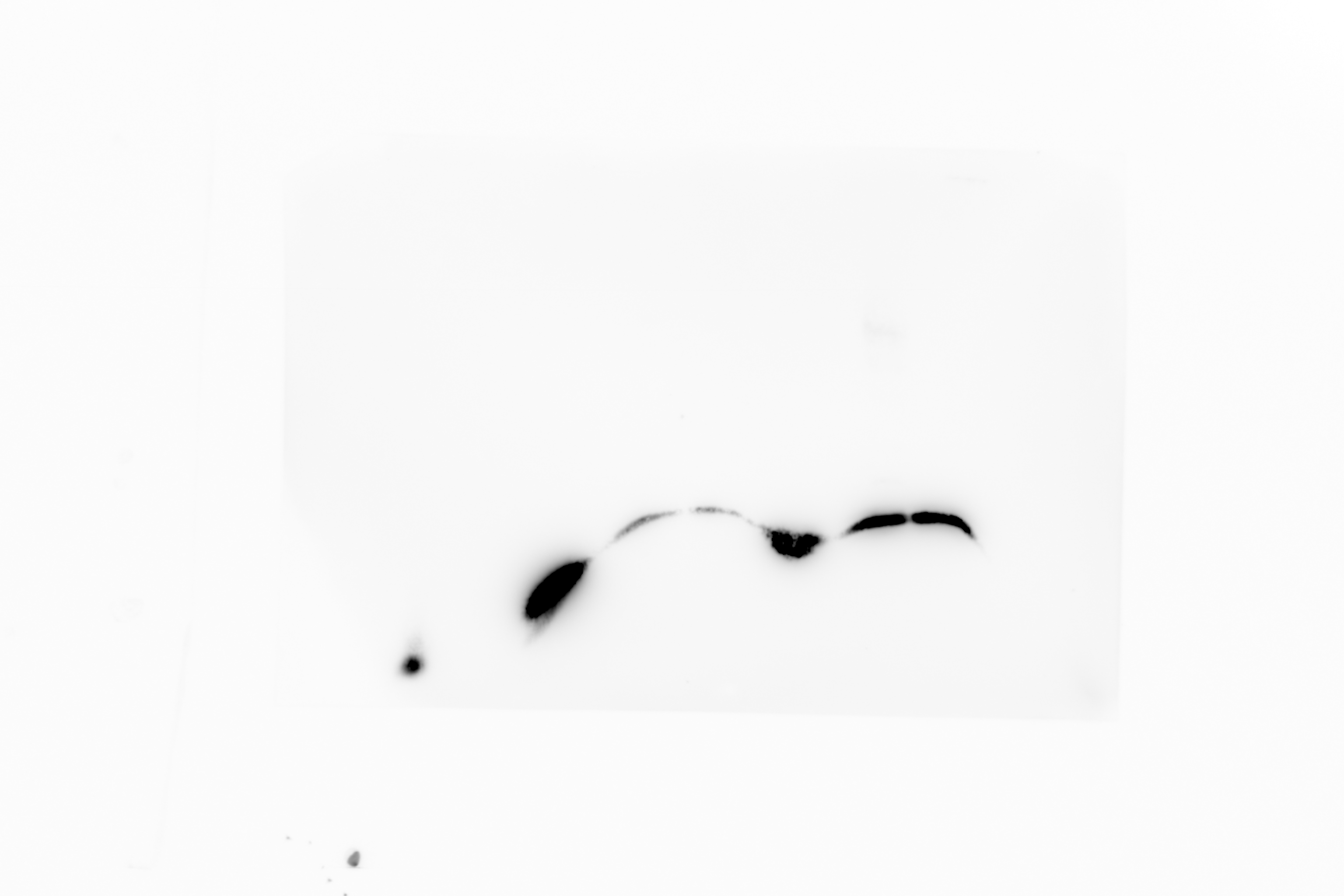

Supplement: Figure 3—figure supplement 2—source data 2. [file elife-99937-fig3-figsupp2-data2.zip › Figure 3- figure supplement 2 source data-EMSA.tif]

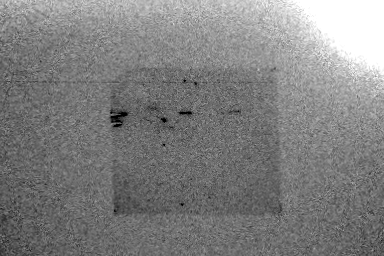

Supplement: Figure 3—figure supplement 2—source data 2. [file elife-99937-fig3-figsupp2-data2.zip › Figure 3- figure supplement 2 source data-WB.tif]

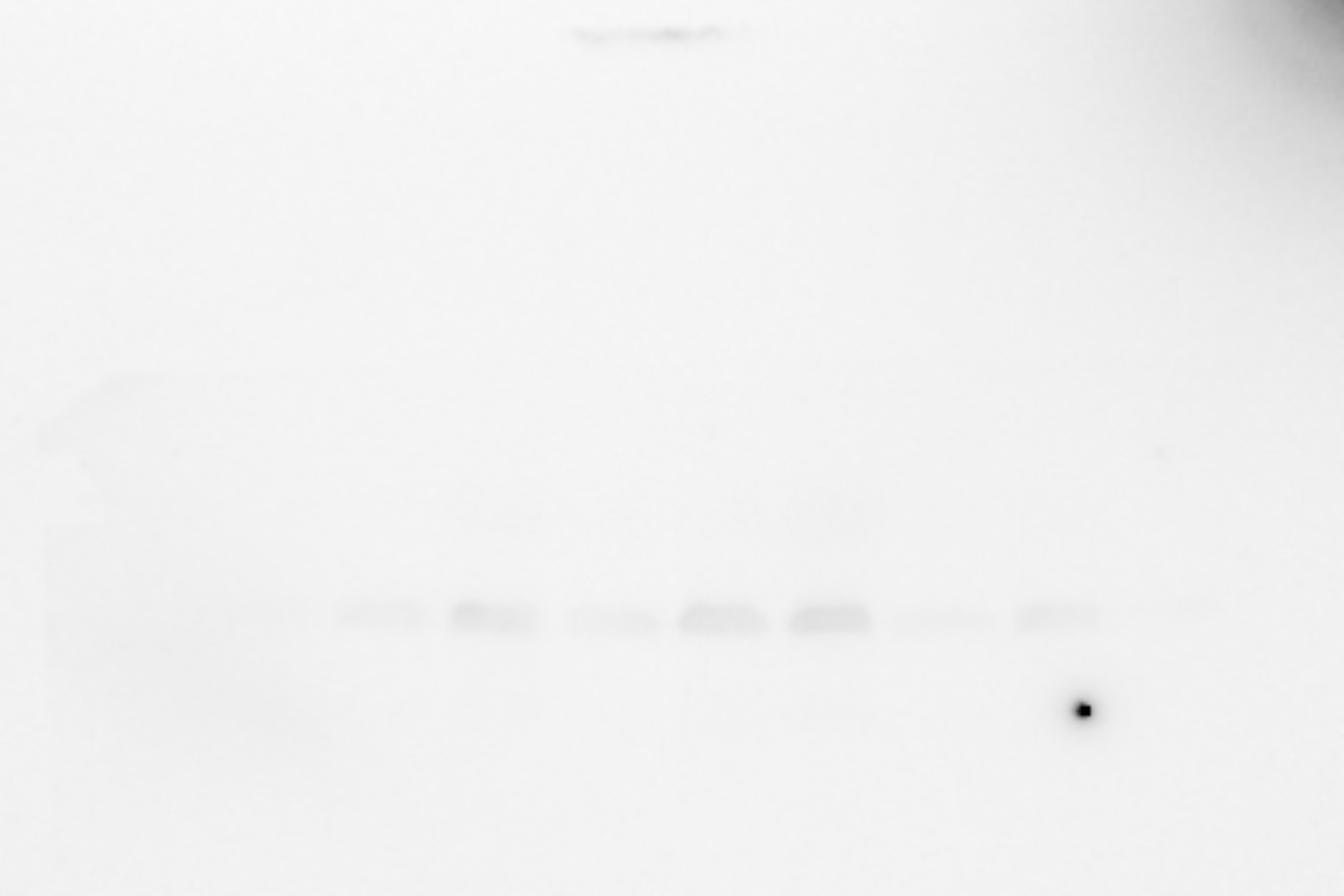

Supplement: Figure 4—source data 2. [file elife-99937-fig4-data2.zip › Figure 4C- source data-WB-GUS.tif]

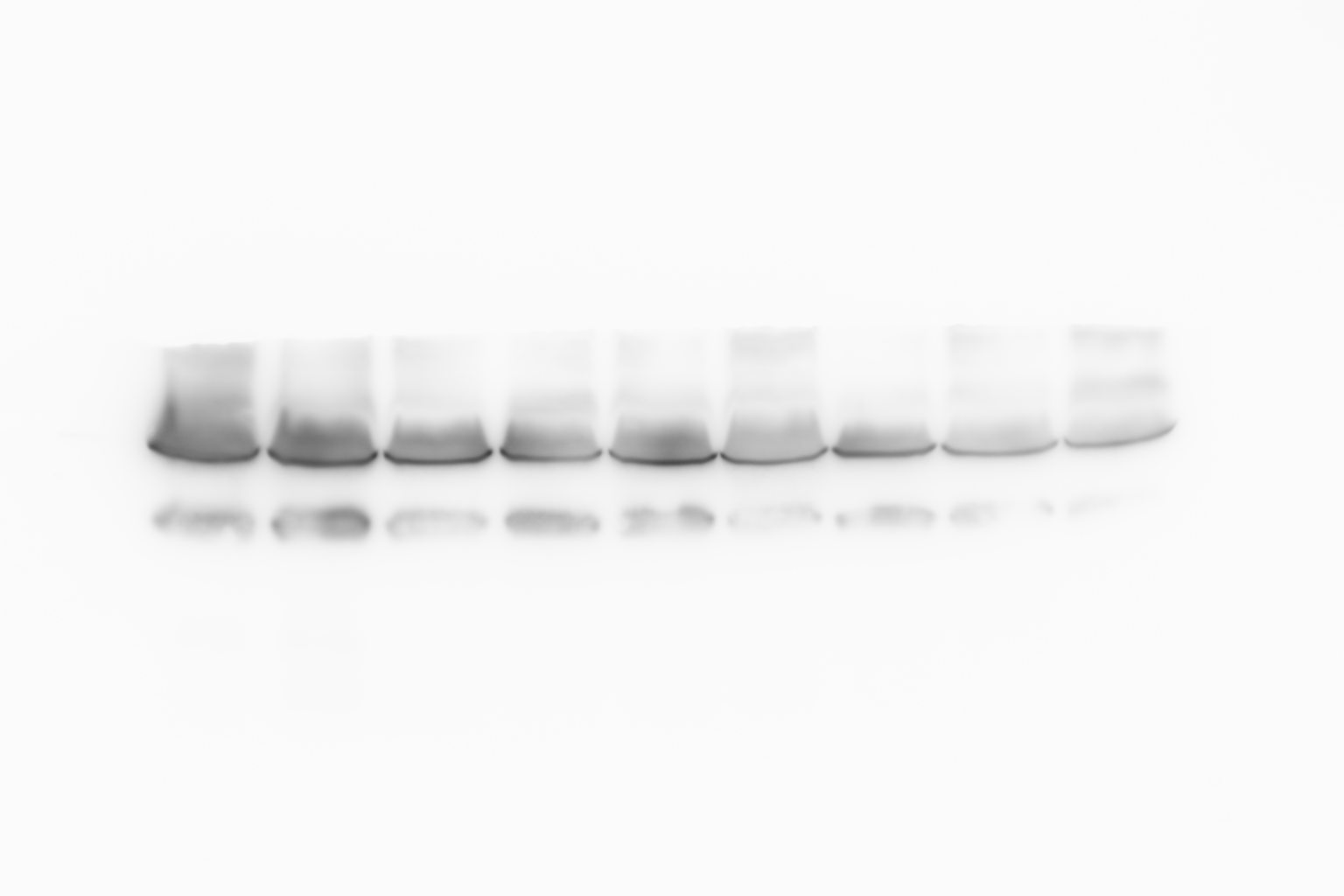

Supplement: Figure 4—source data 2. [file elife-99937-fig4-data2.zip › Figure 4C- source data-WB-Tub.tif]

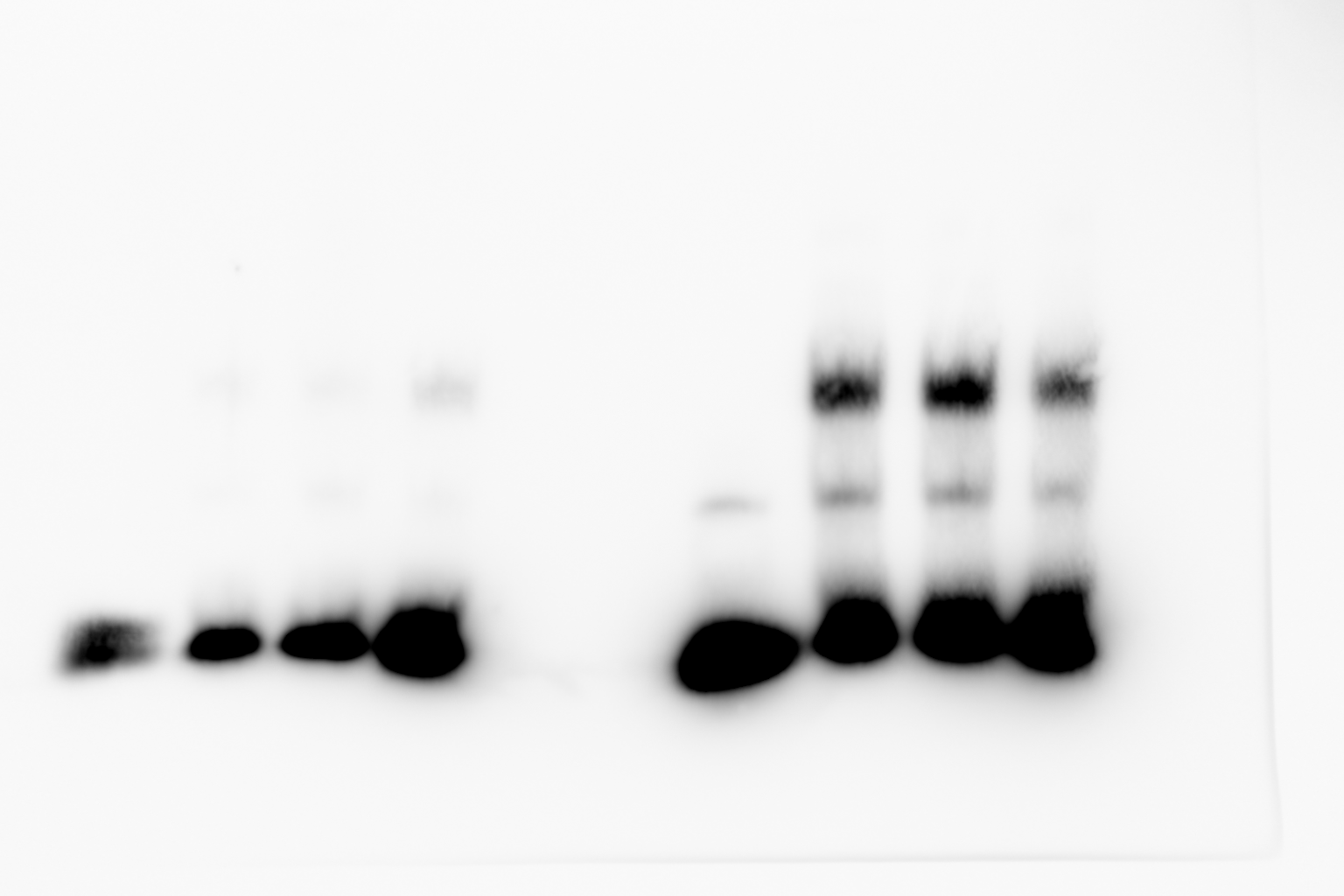

Supplement: Figure 4—source data 2. [file elife-99937-fig4-data2.zip › Figure 4E- source data-EMSA.tif]

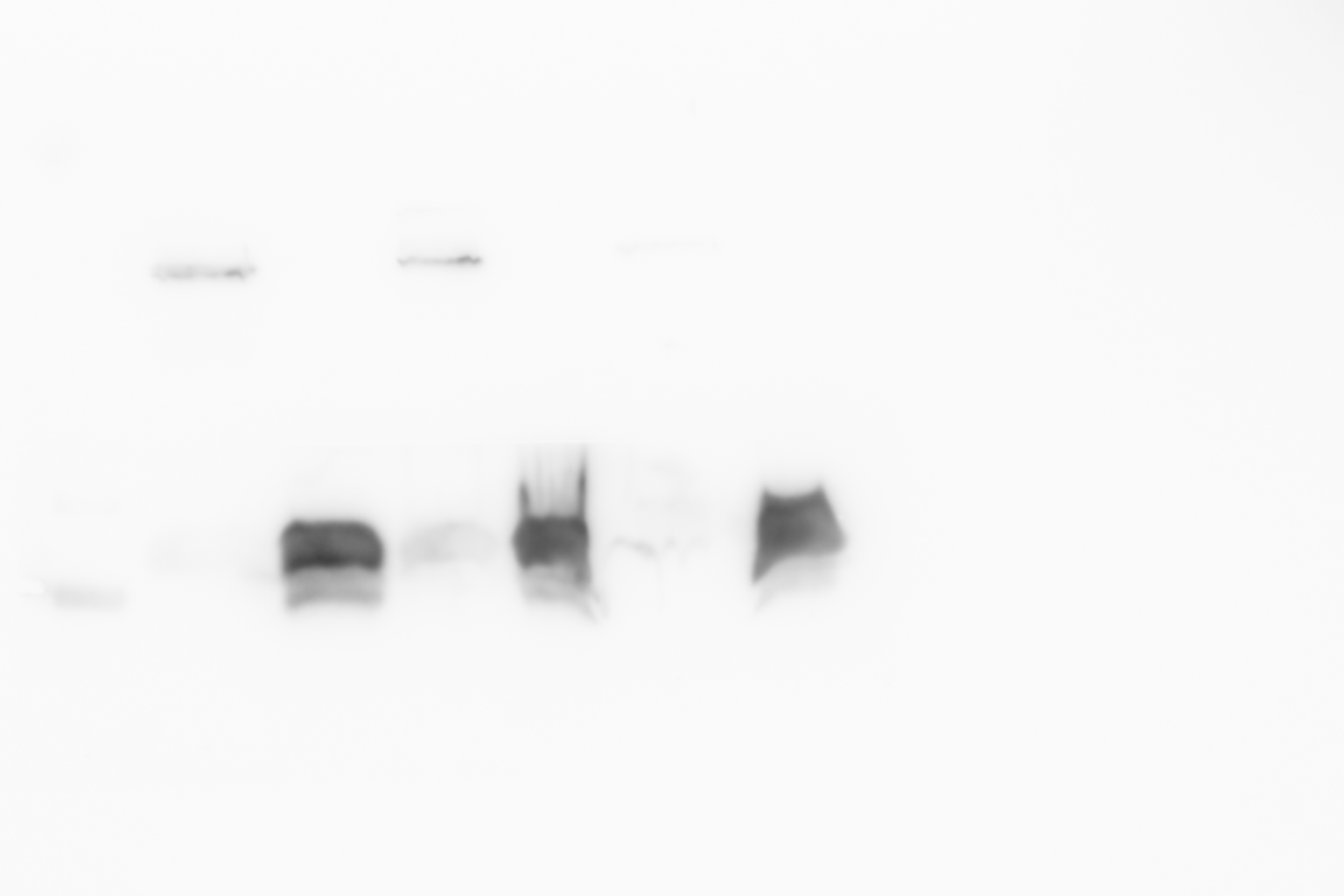

Supplement: Figure 4—source data 2. [file elife-99937-fig4-data2.zip › Figure 4E- source data-WB.tif]

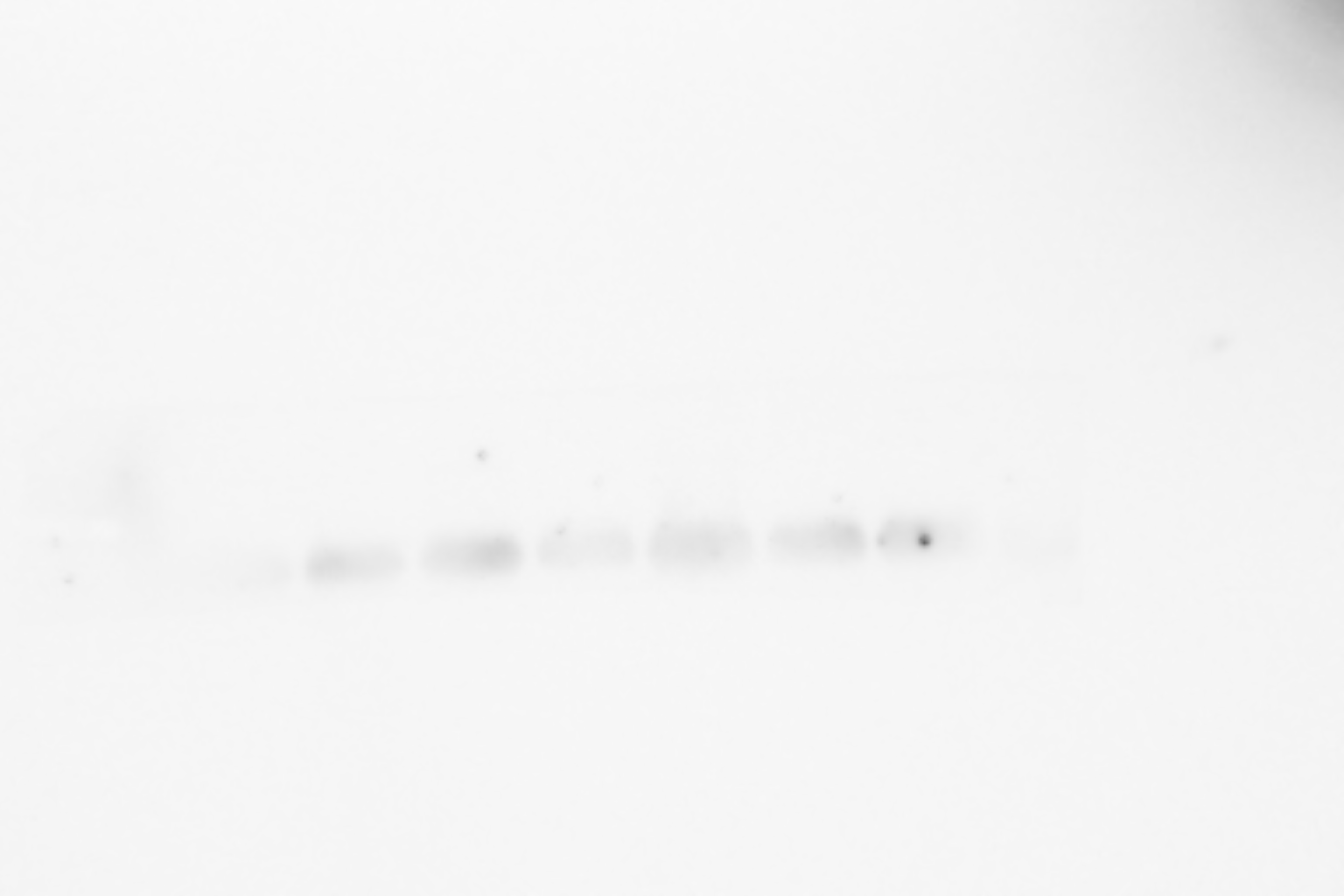

Supplement: Figure 4—source data 2. [file elife-99937-fig4-data2.zip › Figure 4F- source data-WB-GUS.tif]

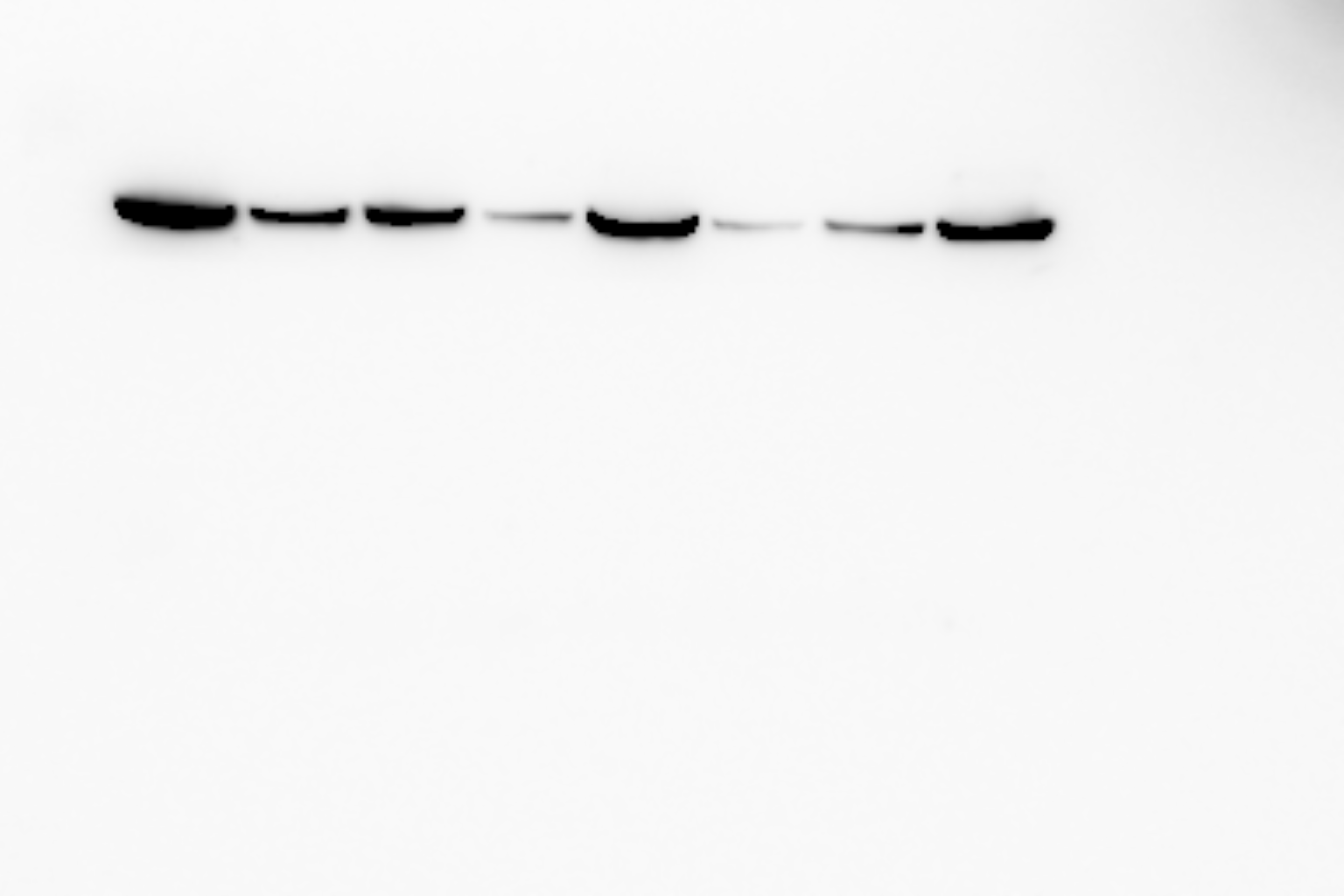

Supplement: Figure 4—source data 2. [file elife-99937-fig4-data2.zip › Figure 4F- source data-WB-Tub.tif]

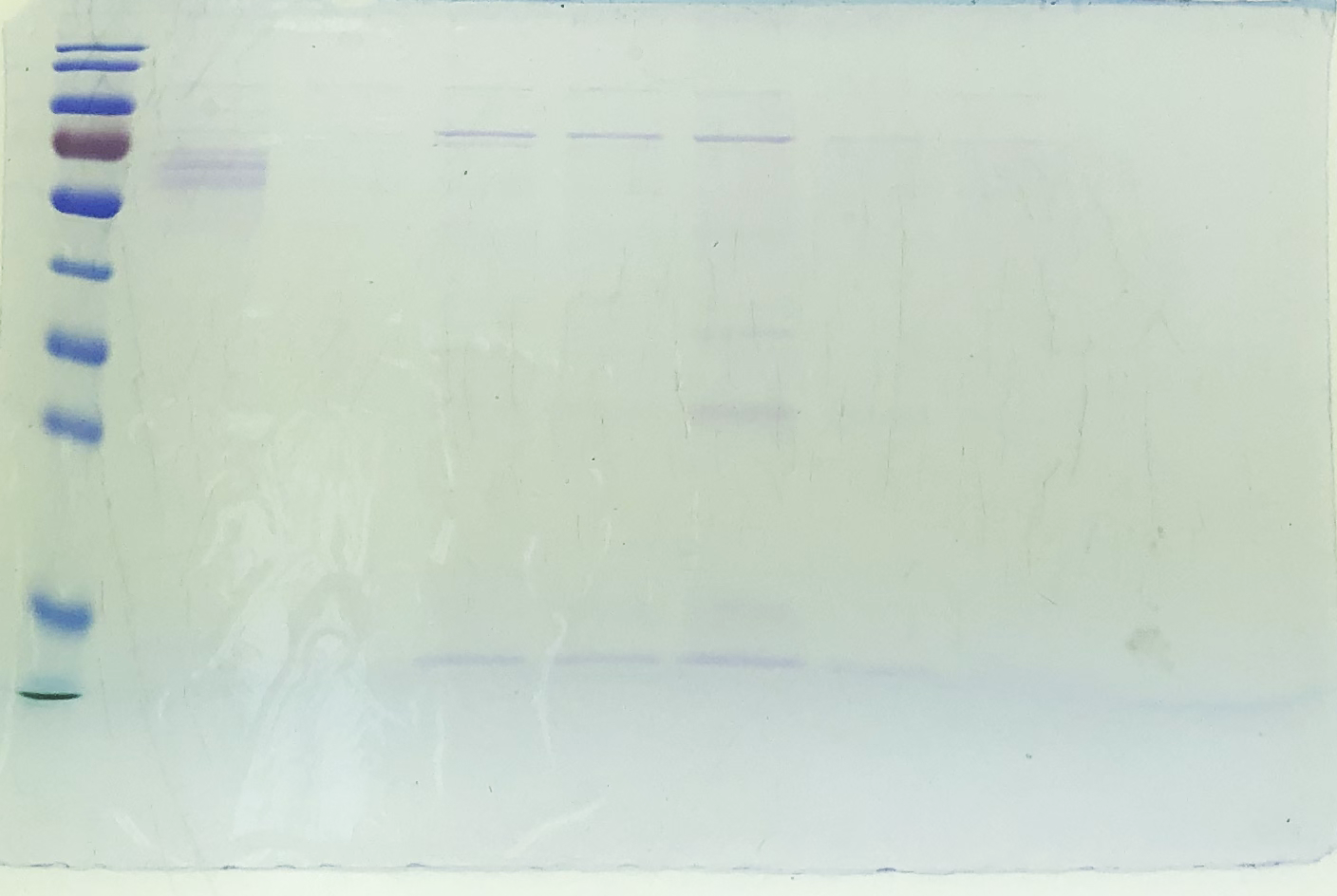

Supplement: Figure 5—source data 2. [file elife-99937-fig5-data2.zip › Figure 5C- source data-CBB.tif]

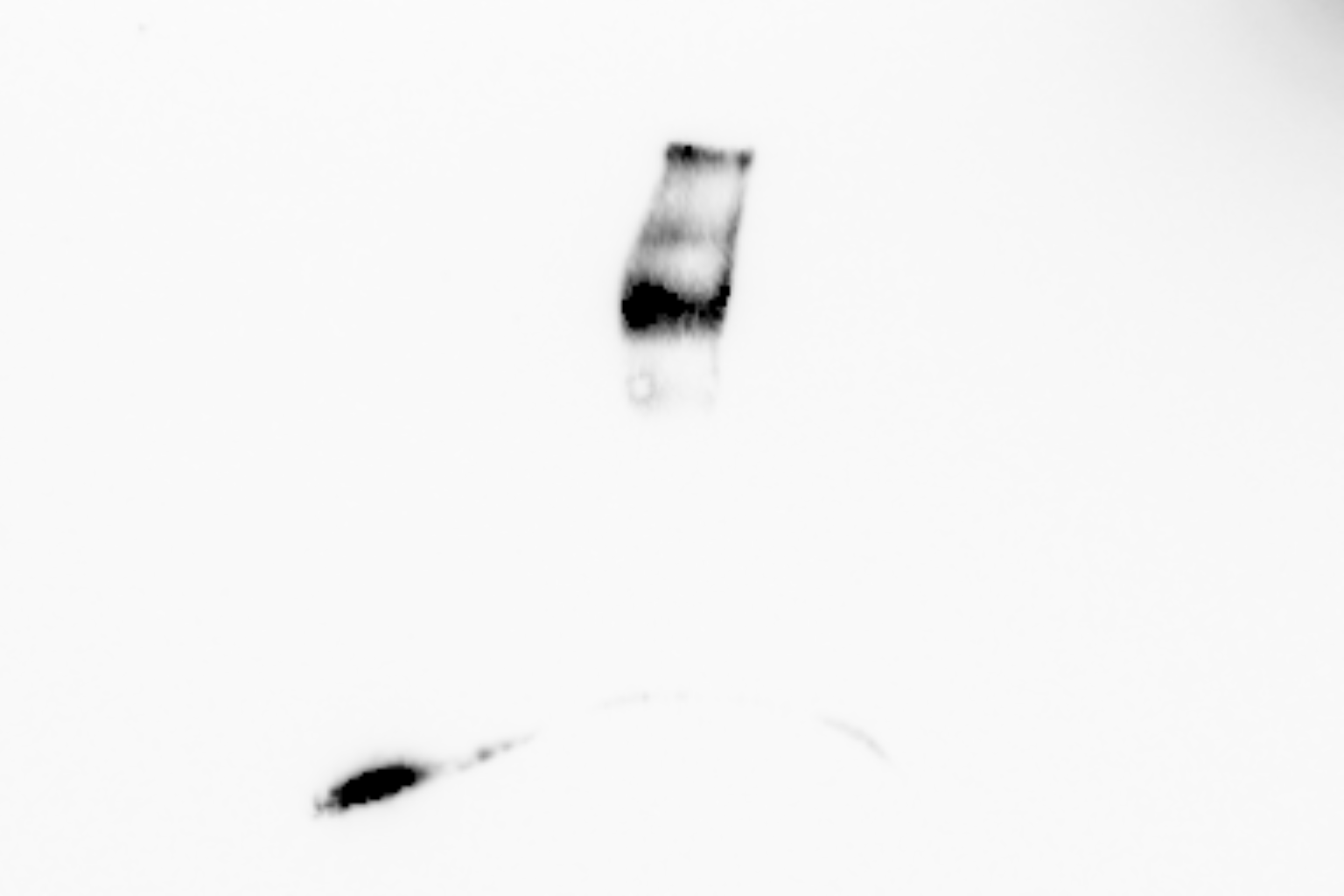

Supplement: Figure 5—source data 2. [file elife-99937-fig5-data2.zip › Figure 5C- source data-EMSA1.tif]

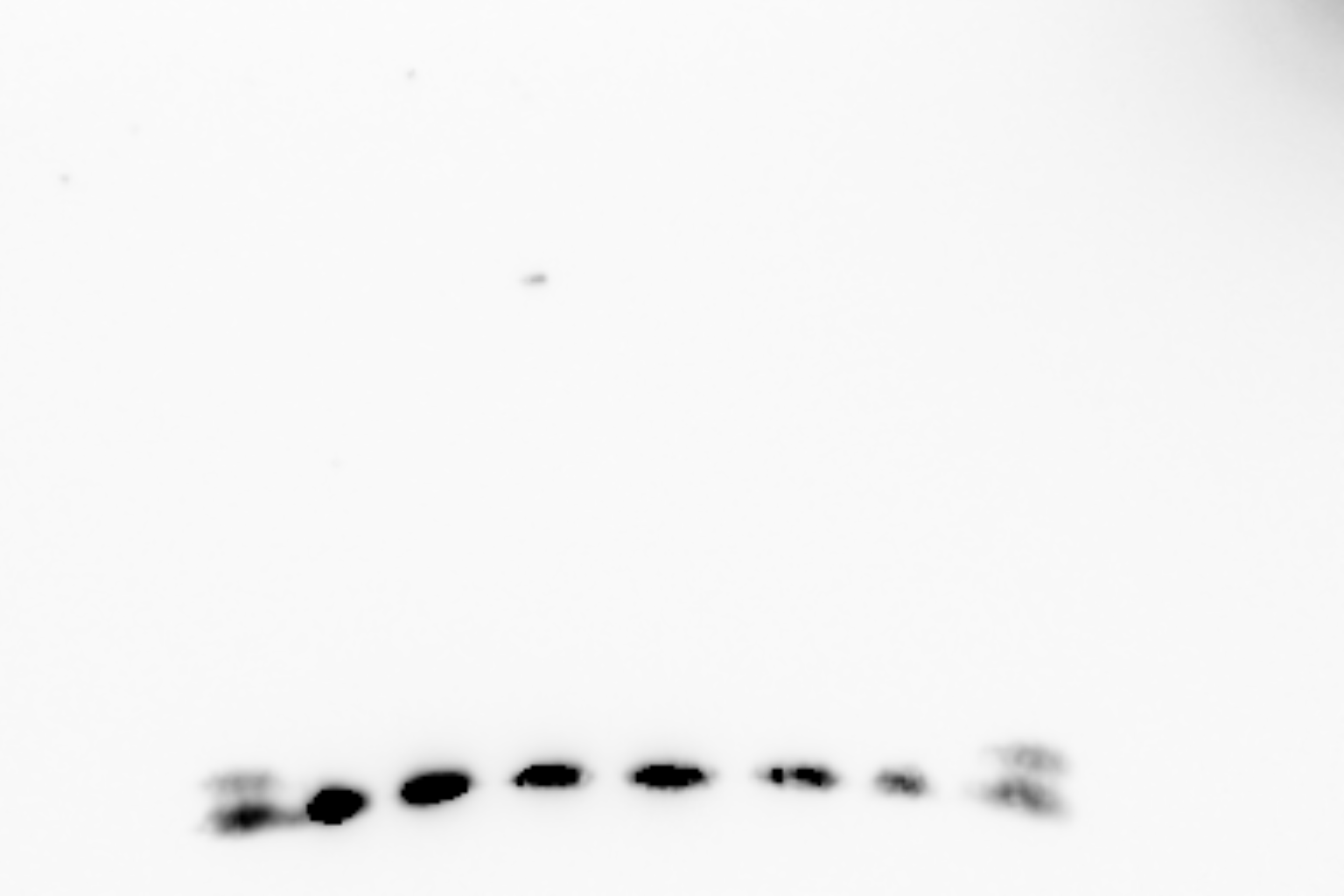

Supplement: Figure 5—source data 2. [file elife-99937-fig5-data2.zip › Figure 5C- source data-EMSA2.tif]

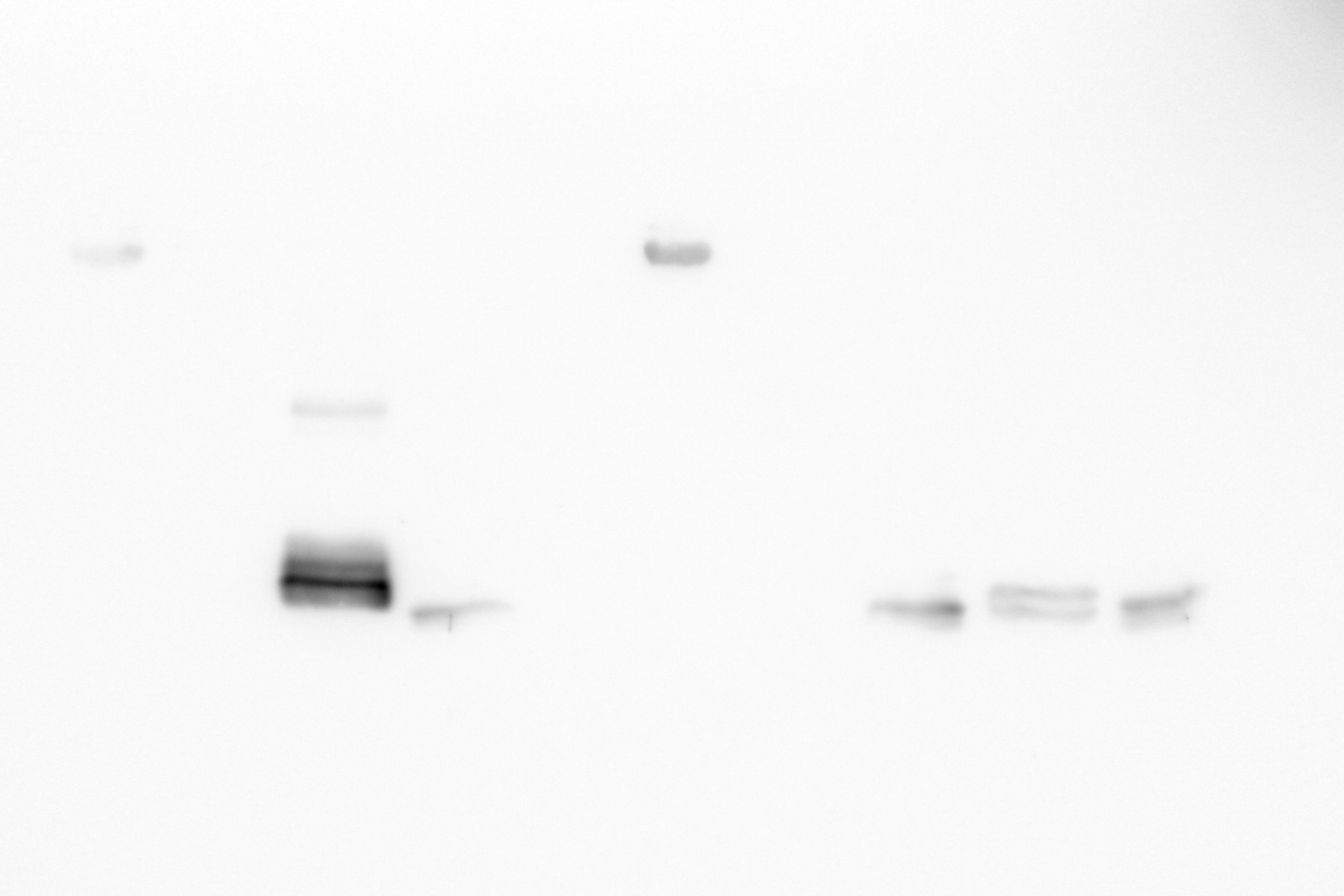

Supplement: Figure 5—source data 2. [file elife-99937-fig5-data2.zip › Figure 5C- source data-WB.tif]

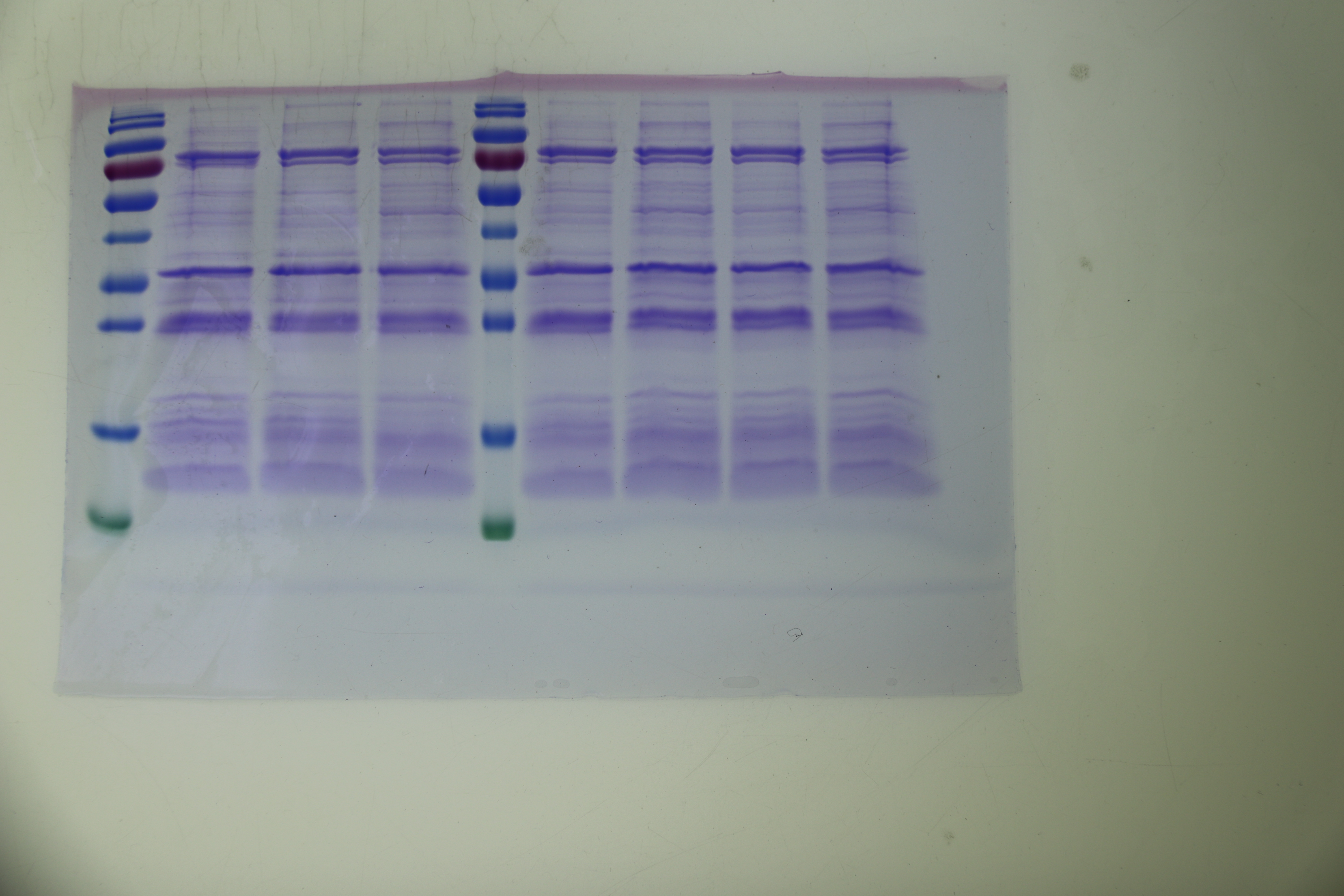

Supplement: Figure 5—figure supplement 2—source data 2. [file elife-99937-fig5-figsupp2-data2.zip › Figure 5-figure supplement 2D source data CBB.JPG]

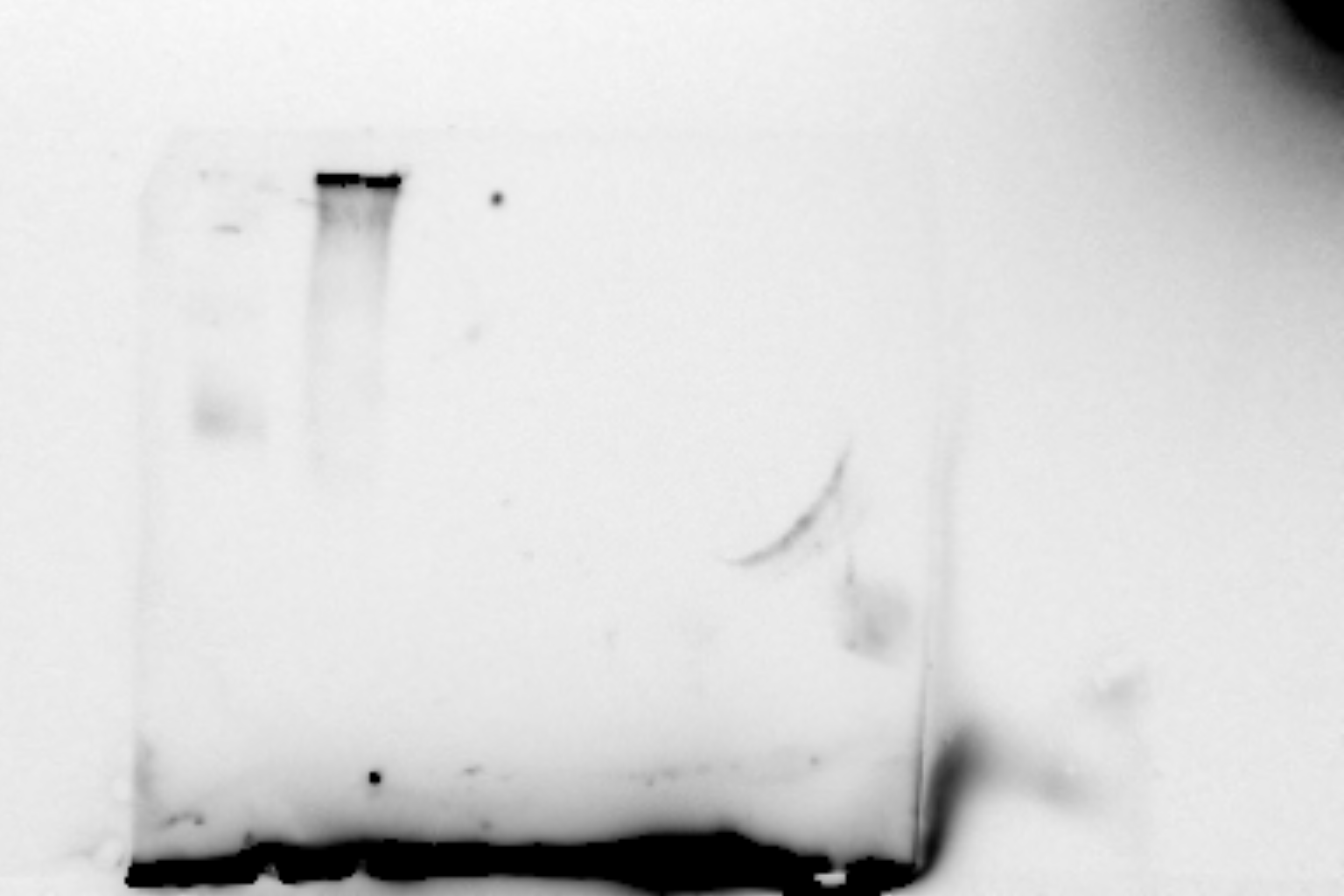

Supplement: Figure 5—figure supplement 2—source data 2. [file elife-99937-fig5-figsupp2-data2.zip › Figure 5-figure supplement 2D source data EMSA-HSE.tif]

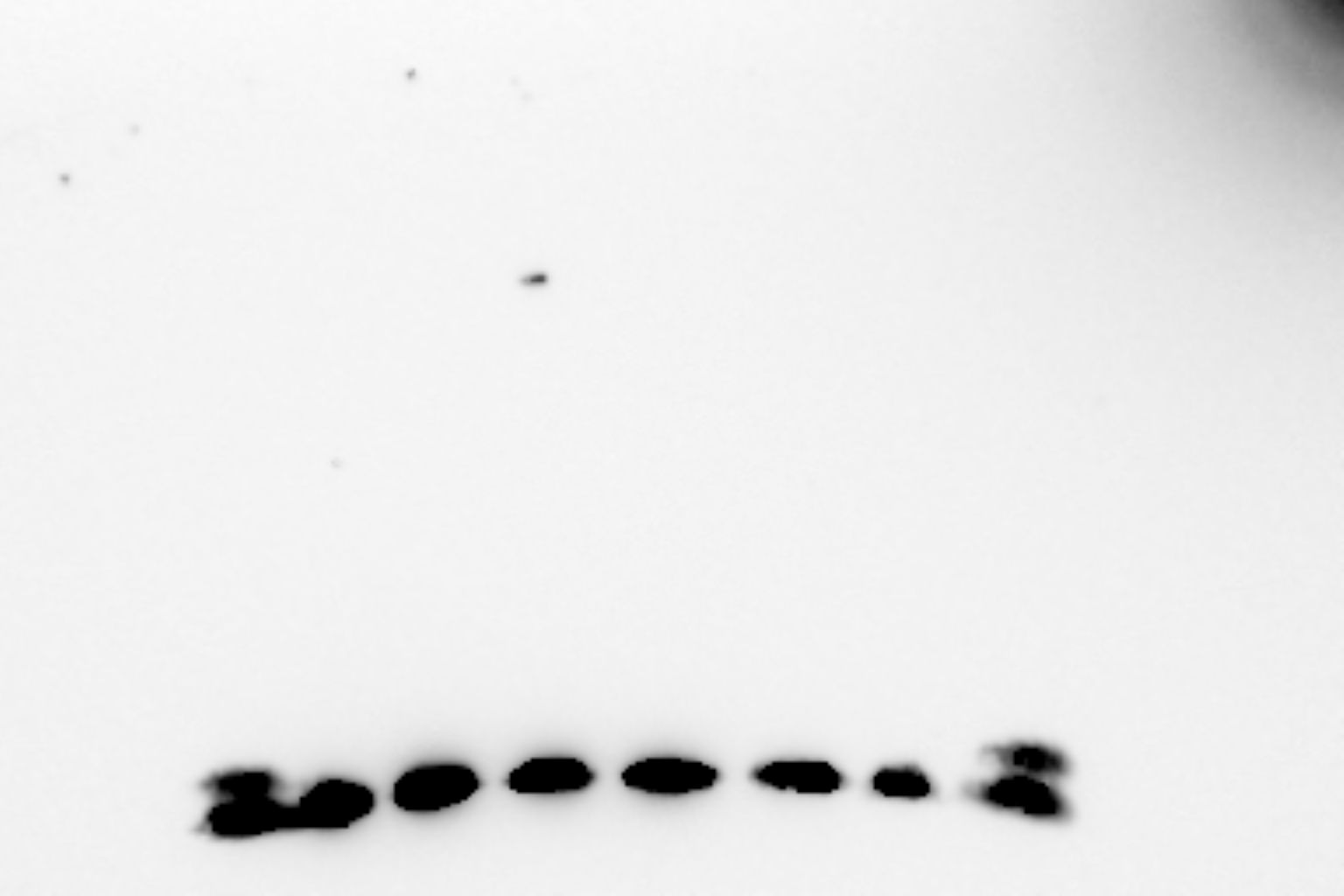

Supplement: Figure 5—figure supplement 2—source data 2. [file elife-99937-fig5-figsupp2-data2.zip › Figure 5-figure supplement 2D source data EMSA-HSEm.tif]

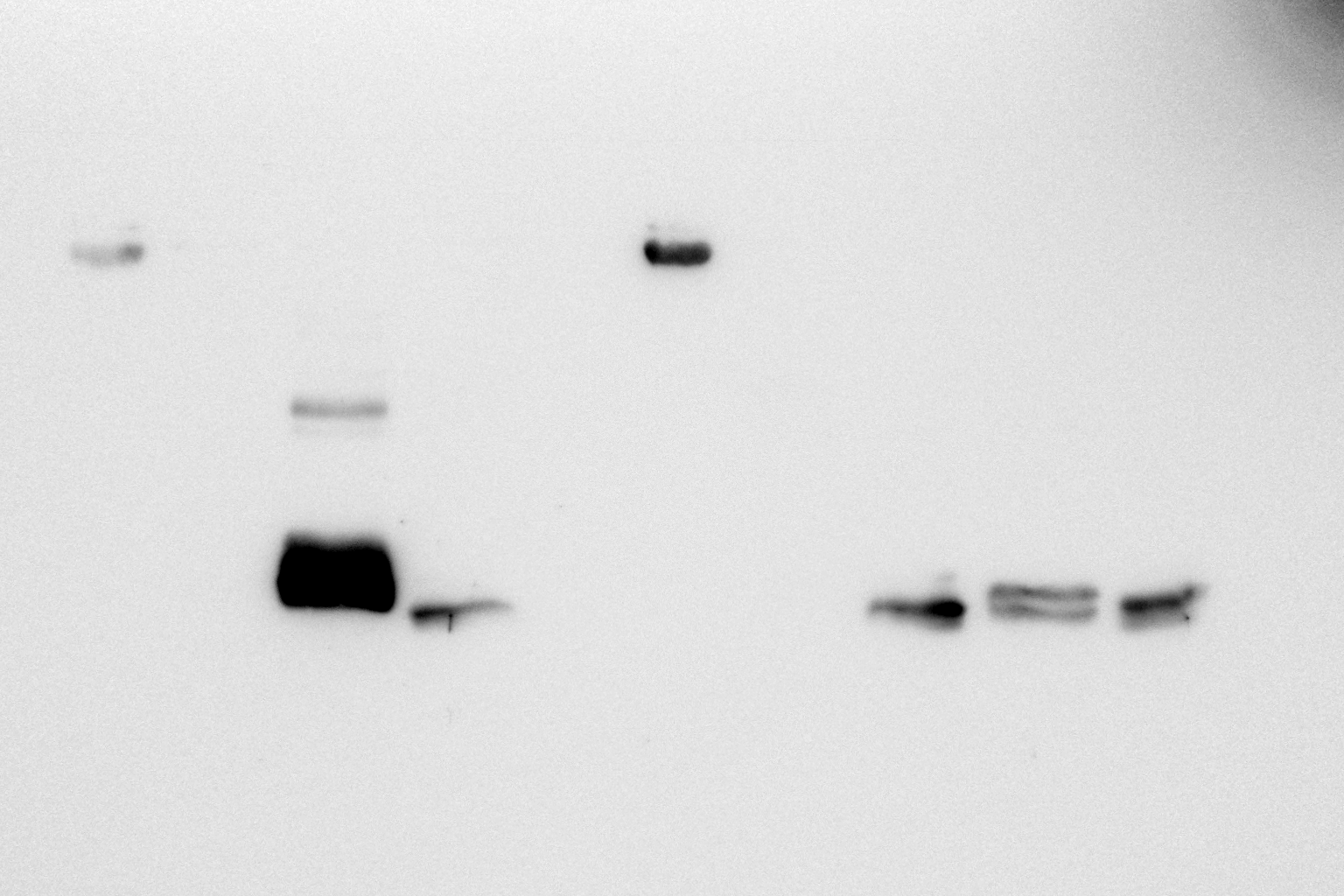

Supplement: Figure 5—figure supplement 2—source data 2. [file elife-99937-fig5-figsupp2-data2.zip › Figure 5-figure supplement 2D source data WB.tif]

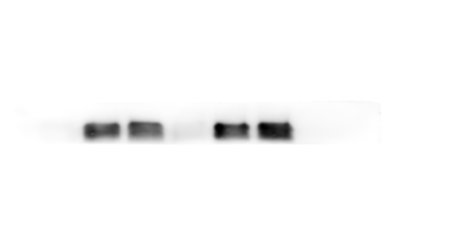

Supplement: Figure 5—figure supplement 2—source data 2. [file elife-99937-fig5-figsupp2-data2.zip › Figure 5-figure supplement 2E source data WB-RFP.tif]

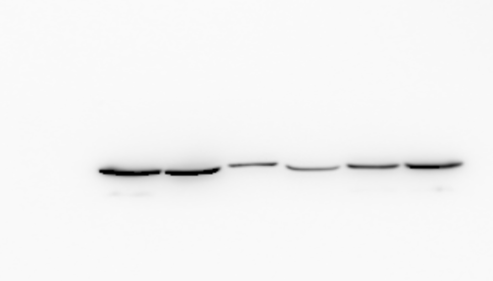

Supplement: Figure 5—figure supplement 2—source data 2. [file elife-99937-fig5-figsupp2-data2.zip › Figure 5-figure supplement 2E source data WB-tub.tif]

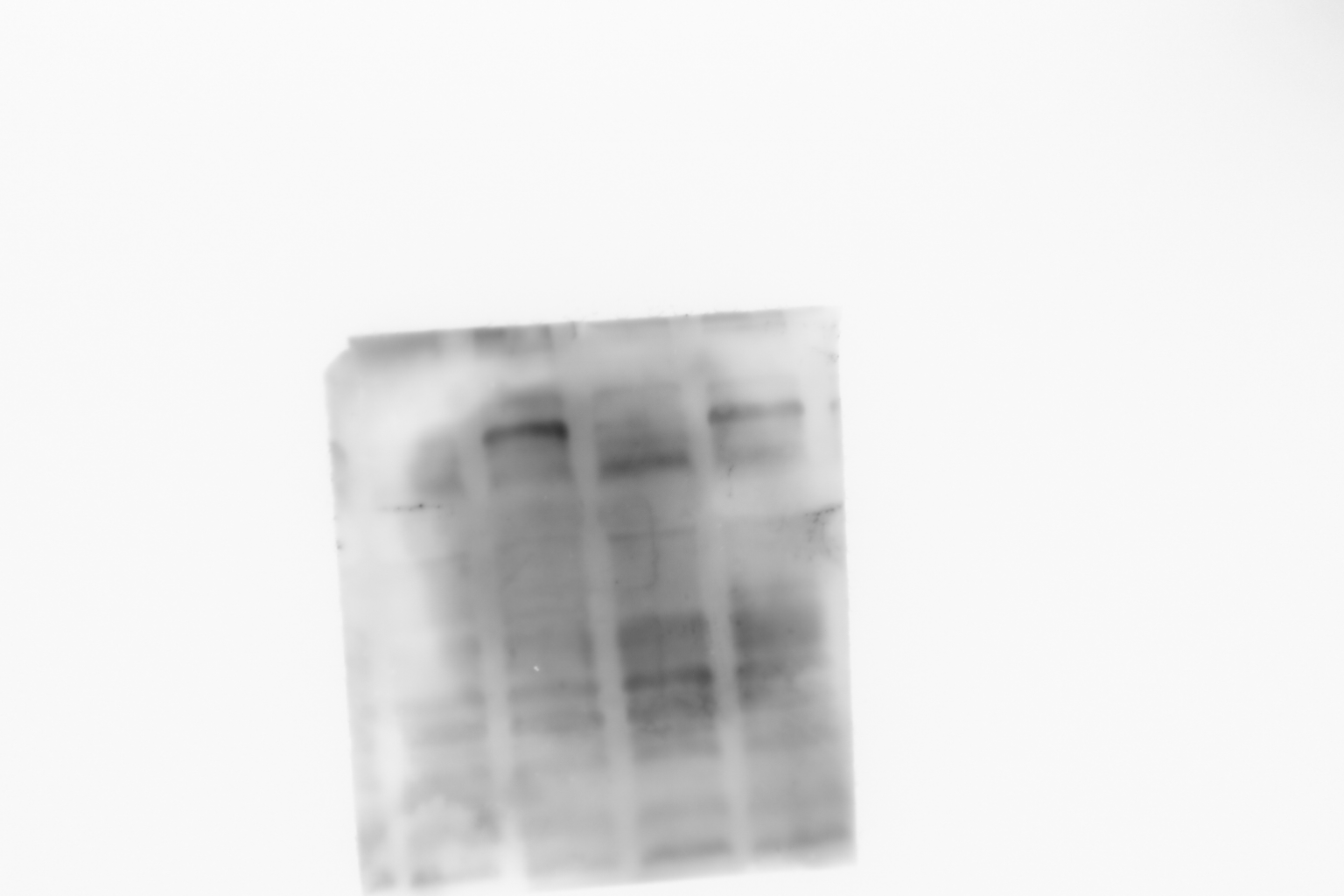

Supplement: Figure 6—figure supplement 1—source data 2. [file elife-99937-fig6-figsupp1-data2.zip › Figure 6- figure supplement 1 source data -rfp.tif]

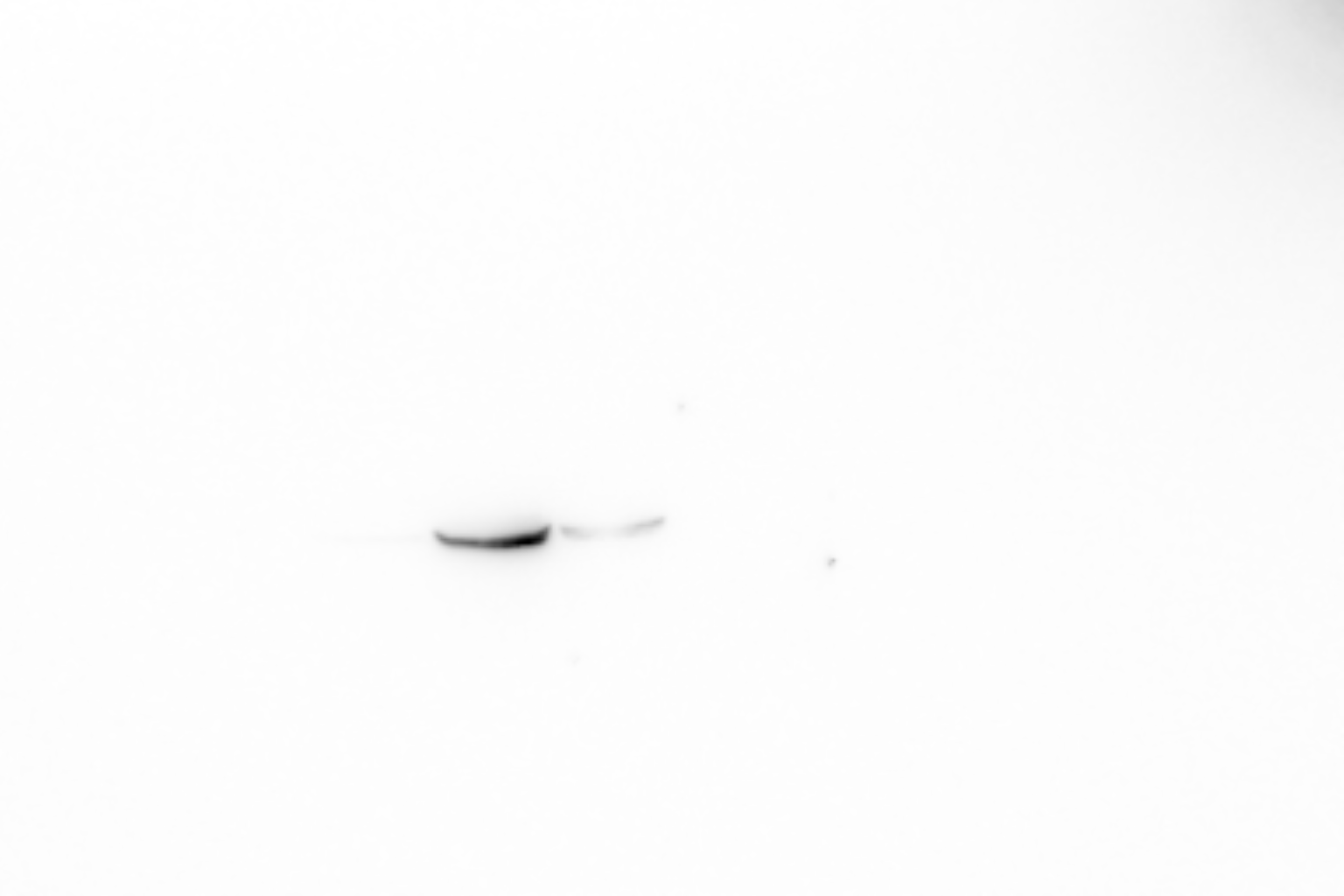

Supplement: Figure 6—figure supplement 1—source data 2. [file elife-99937-fig6-figsupp1-data2.zip › Figure 6- figure supplement 1 source data -tub.tif]

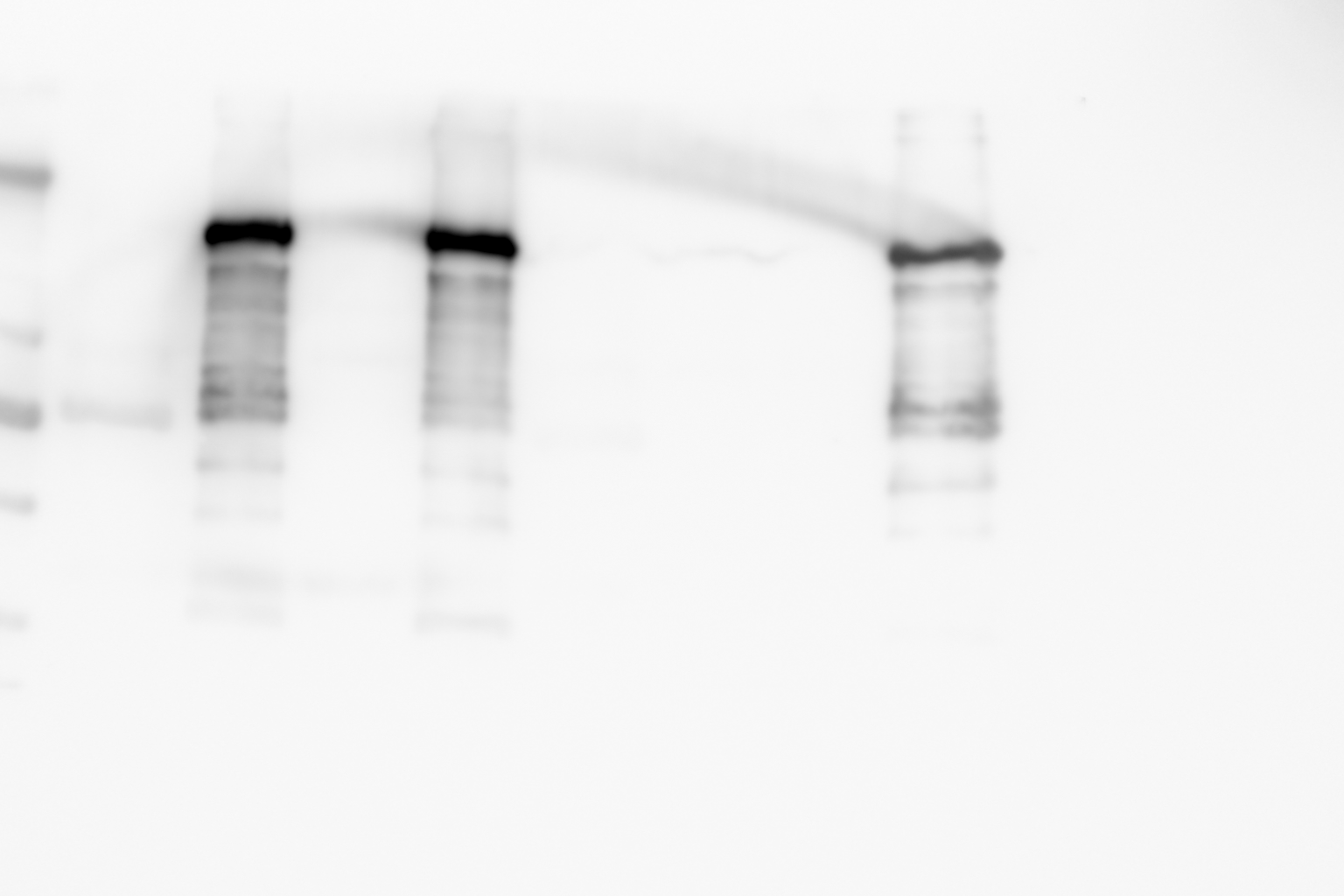

Supplement: Figure 7—source data 2. [file elife-99937-fig7-data2.zip › Figure 7B source data -his.tif]

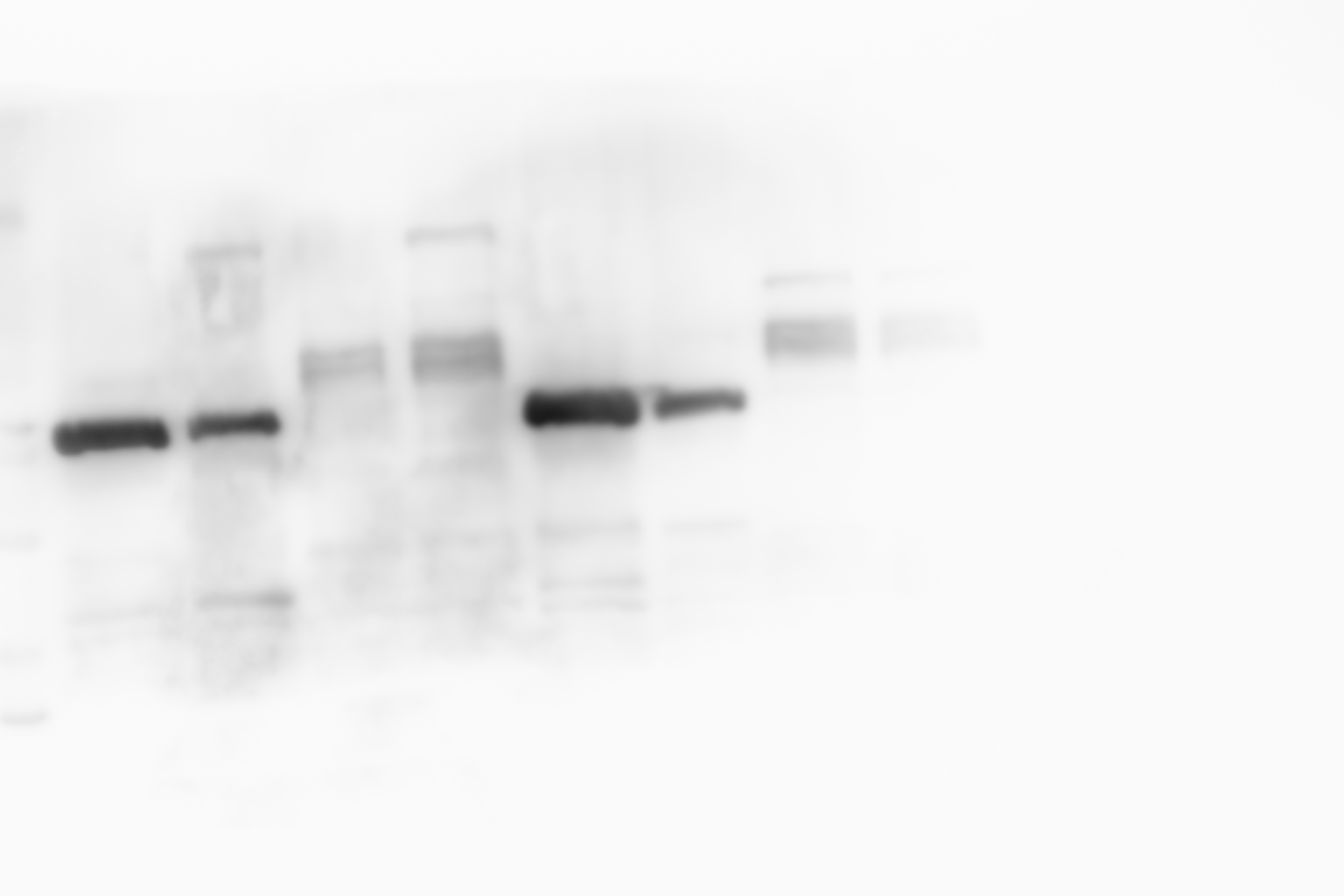

Supplement: Figure 7—source data 2. [file elife-99937-fig7-data2.zip › Figure 7B source data-GST.tif]

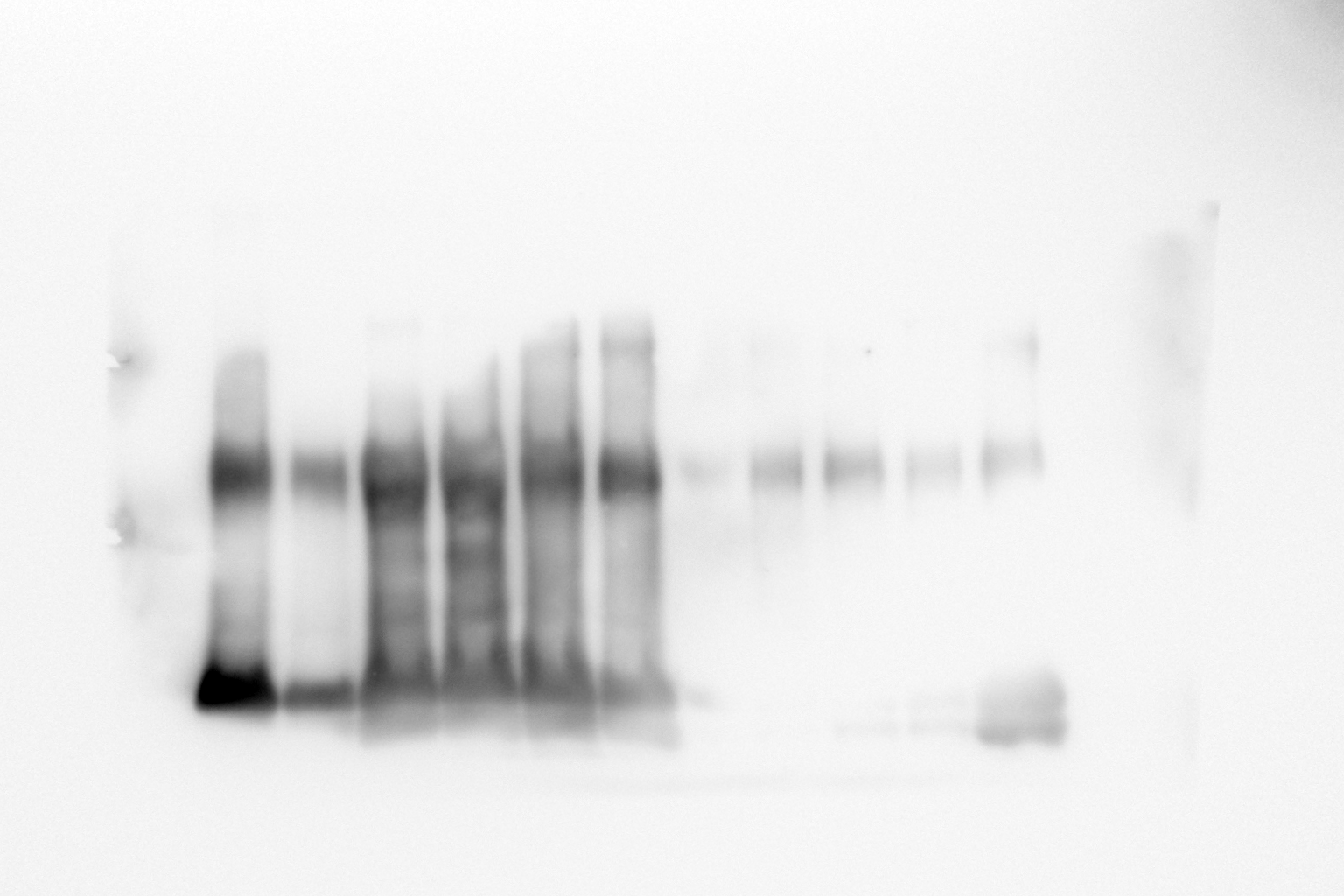

Supplement: Figure 7—source data 2. [file elife-99937-fig7-data2.zip › Figure 7D source data -input.tif]

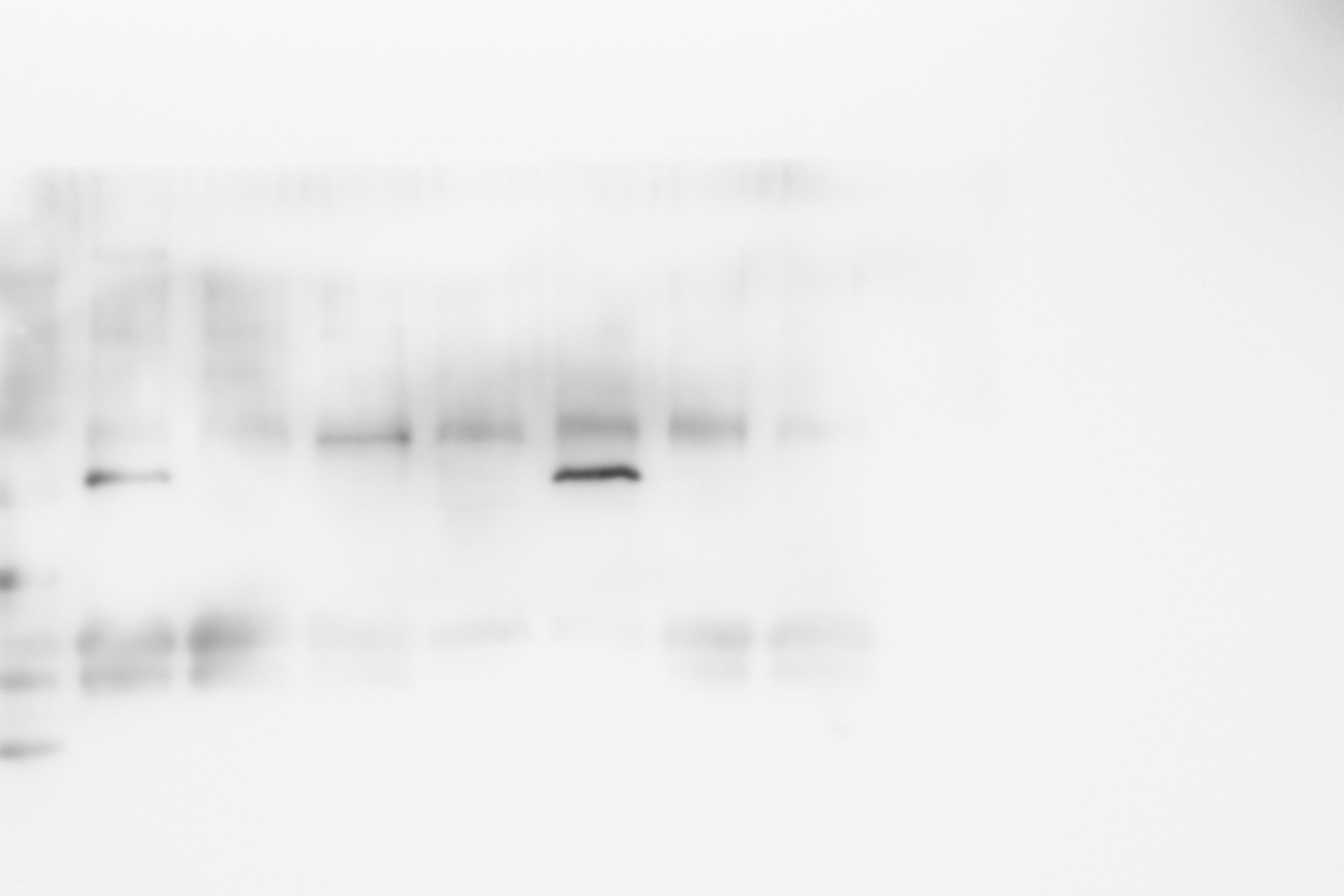

Supplement: Figure 7—source data 2. [file elife-99937-fig7-data2.zip › Figure 7D source data -pulldown.tif]
