# Supplementary material for: Short heat shock factor A2 regulates heat resistance and growth balance in Arabidopsis
Source: eLife. 2025 Nov 3;13:RP99937. doi: 10.7554/eLife.99937 (PMC12582567; doi:10.7554/eLife.99937)
Supplement: Figure 3—source data 1. [file elife-99937-fig3-data1.pdf]

Source data 1. PDF file containing original EMSAs and western blot for **Figure 3C and D**, showing the relevant bands boxed and labelled.

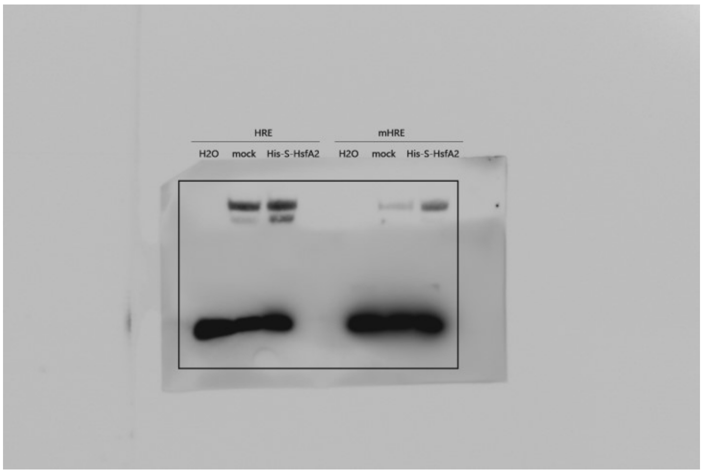

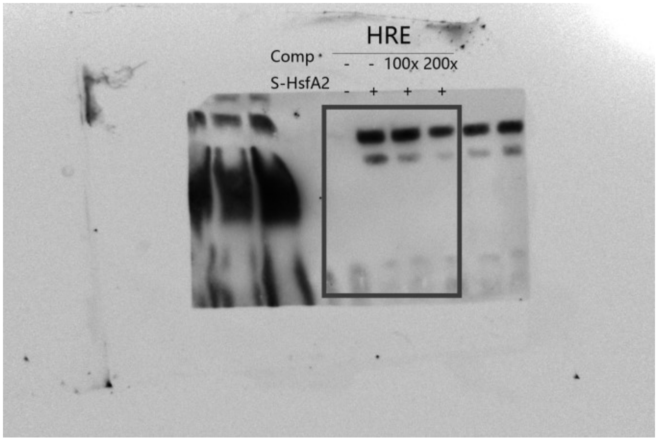

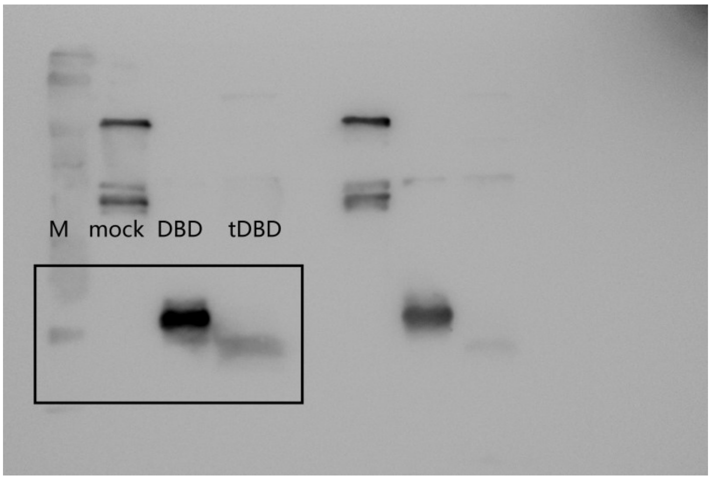

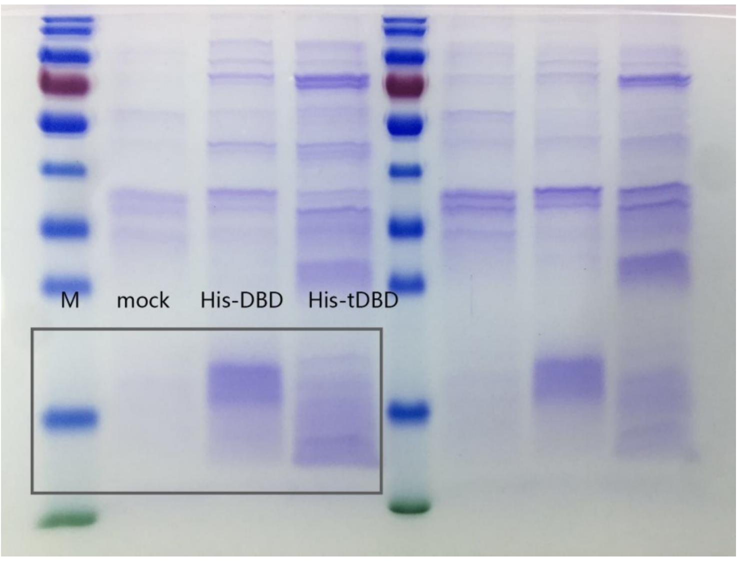
M
mock
His-DBD
His-tDBD

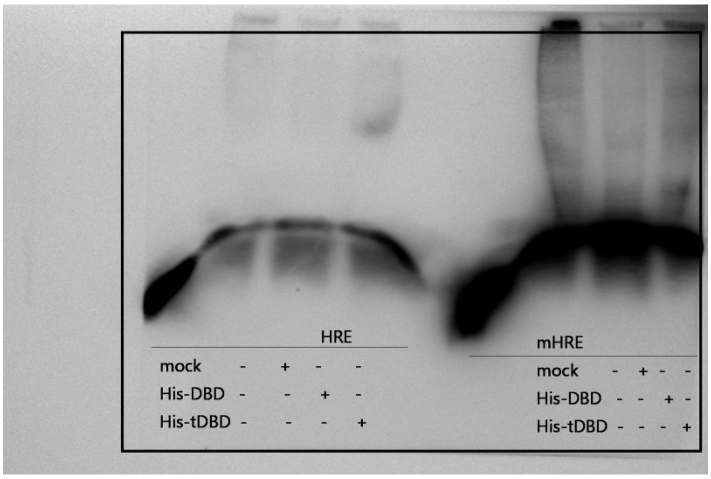
HRE
mock - + - -
His-DBD - - + -
His-tDBD - - - +
mHRE
mock - + - -
His-DBD - - + -
His-tDBD - - - +

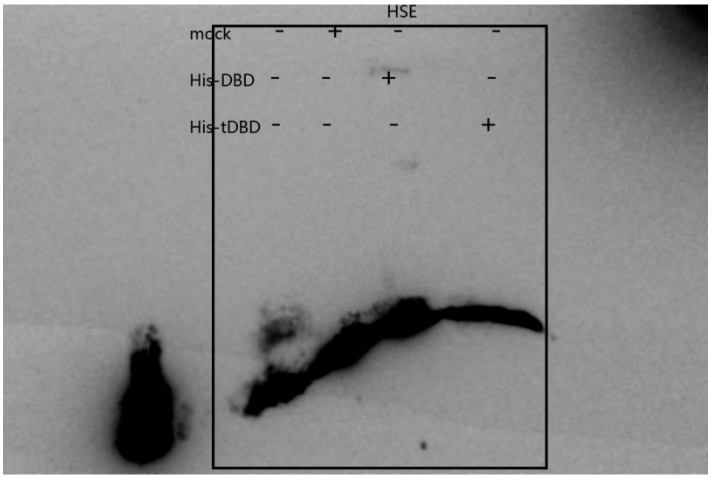
HSE
mock - + - -
His-DBD - - + -
His-tDBD - - - +

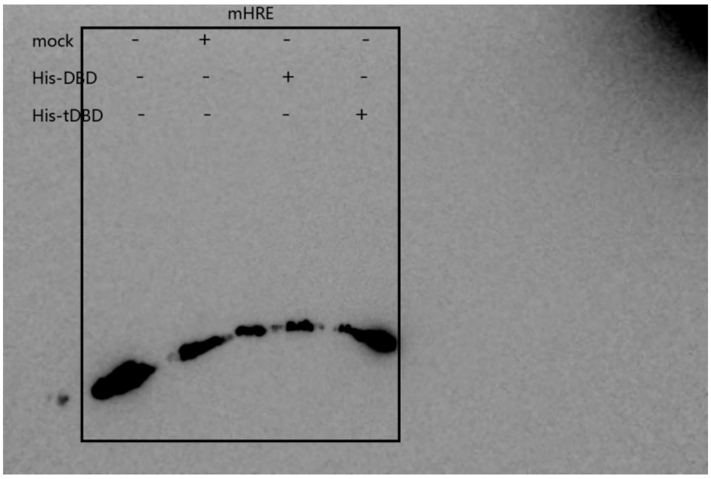
mHRE
mock - + - -
His-DBD - - + -
His-tDBD - - - +
