# Supplementary material for: Short heat shock factor A2 regulates heat resistance and growth balance in Arabidopsis
Source: eLife. 2025 Nov 3;13:RP99937. doi: 10.7554/eLife.99937 (PMC12582567; doi:10.7554/eLife.99937)
Supplement: Figure 4—source data 1. [file elife-99937-fig4-data1.pdf]

Source data 1. PDF file containing original EMSA and western blot for **Figure 4C, E, and F**, showing the relevant bands boxed and labelled.

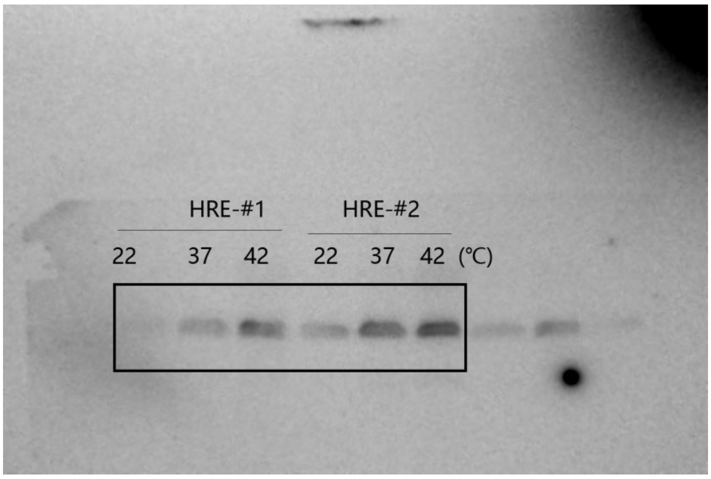

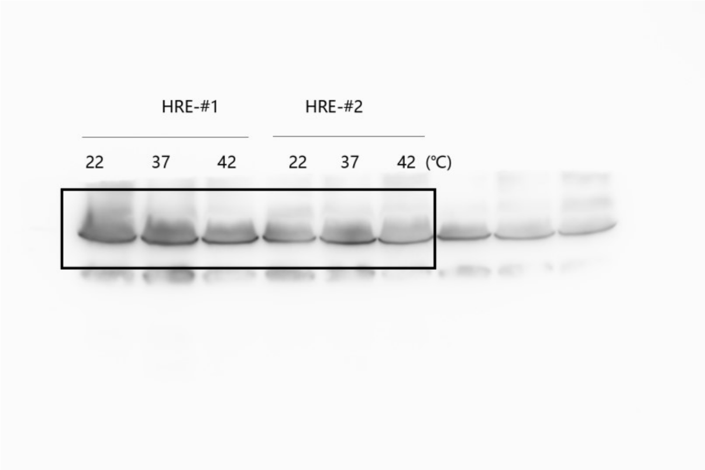

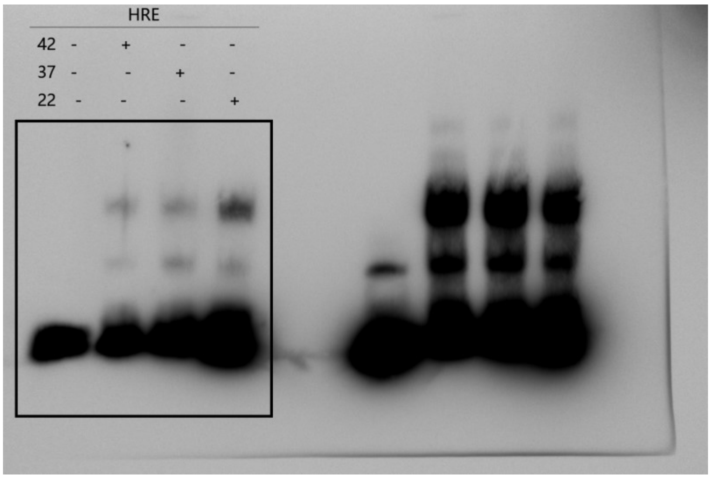

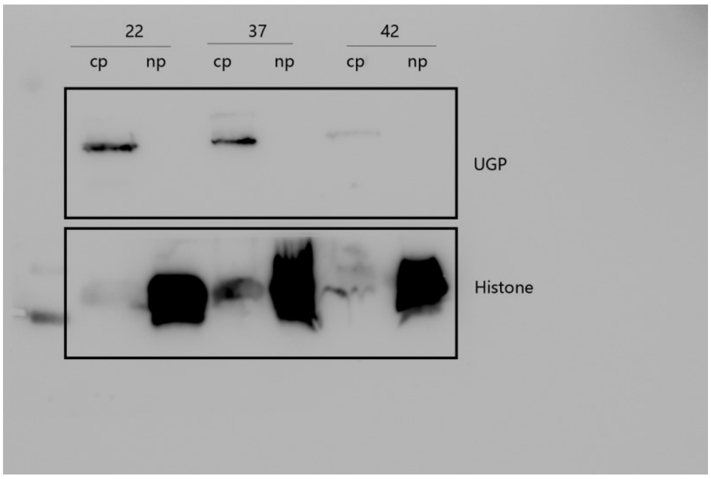
22
37
42
cp
np
cp
np
cp
np
UGP
Histone

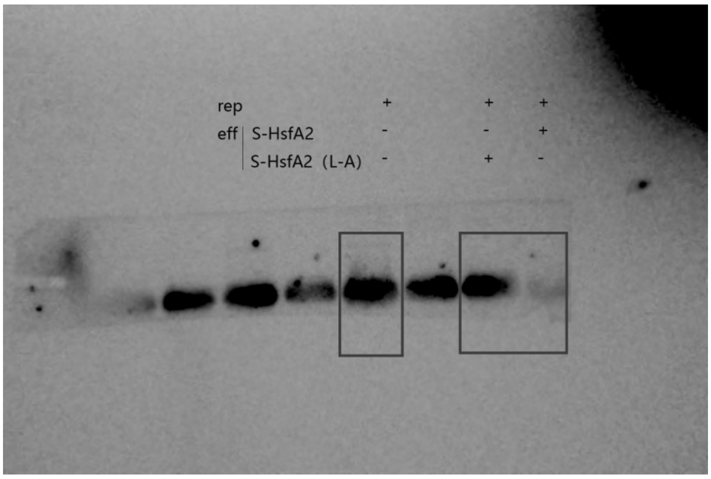
rep + + +
eff S-HsfA2 - - +
S-HsfA2 (L-A) - + -

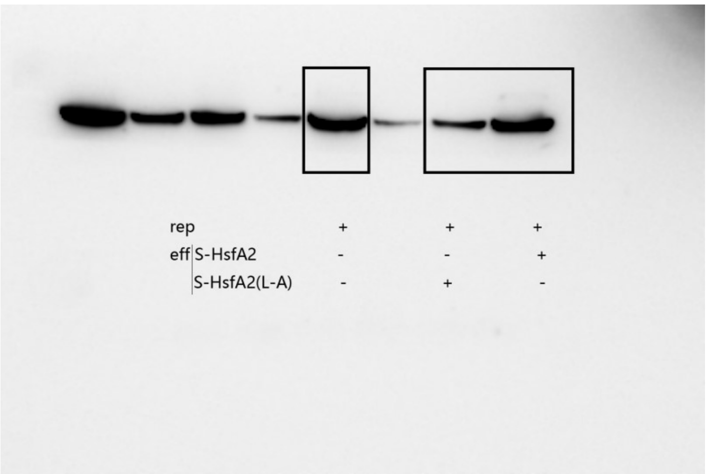
rep + + +
eff S-HsfA2 - - +
S-HsfA2(L-A) - + -
