# Supplementary material for: Short heat shock factor A2 regulates heat resistance and growth balance in Arabidopsis
Source: eLife. 2025 Nov 3;13:RP99937. doi: 10.7554/eLife.99937 (PMC12582567; doi:10.7554/eLife.99937)
Supplement: Figure 5—source data 1. [file elife-99937-fig5-data1.pdf]

**Source data 1**. PDF file containing original EMSA and western blot for **Figure 5C**, showing the relevant bands boxed and labelled.

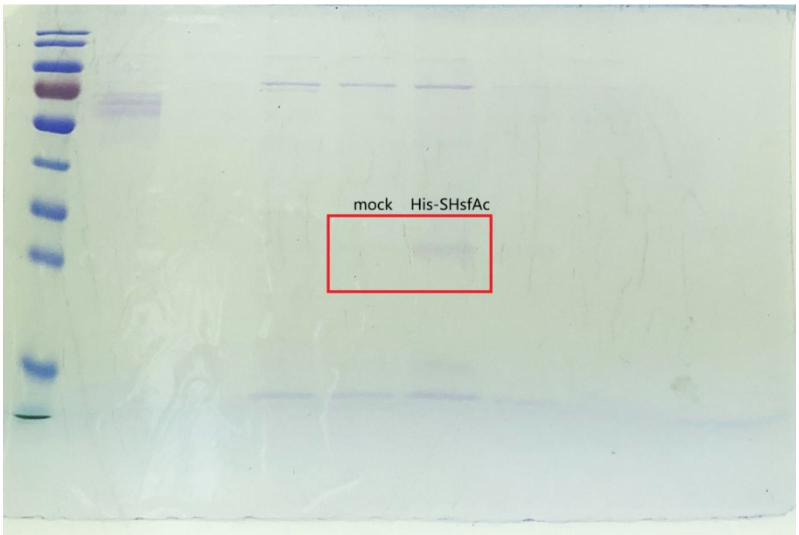


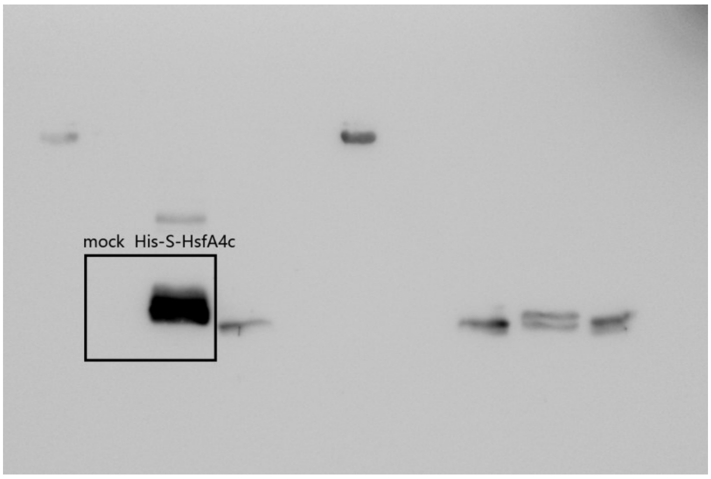


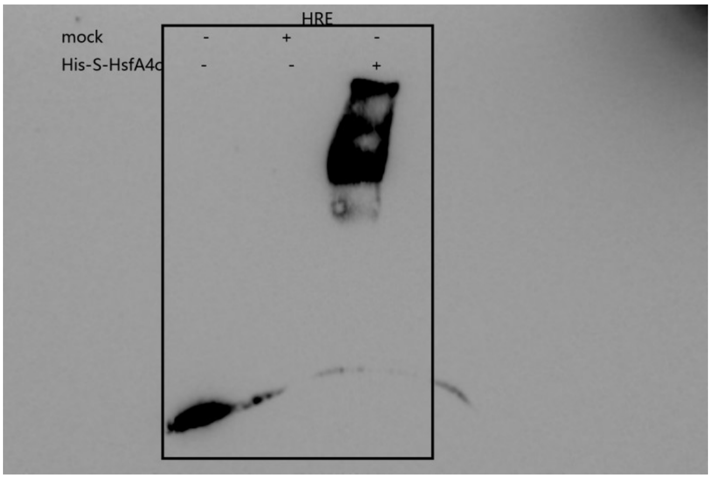

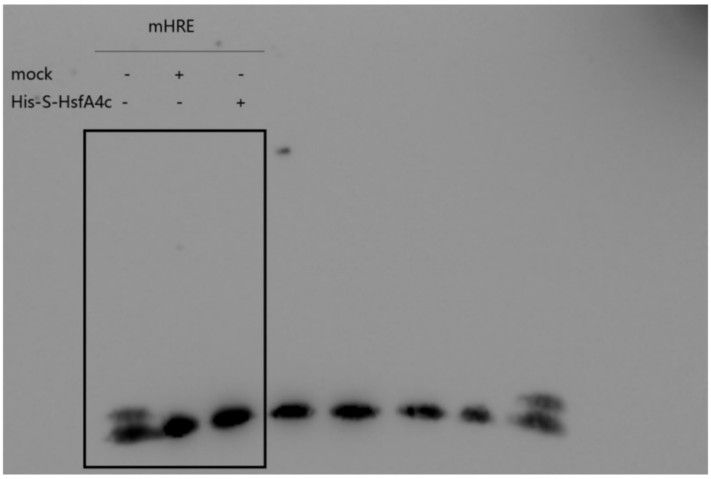
mHRE
mock - + -
His-S-HsfA4c - - +
