# Supplementary material for: Short heat shock factor A2 regulates heat resistance and growth balance in Arabidopsis
Source: eLife. 2025 Nov 3;13:RP99937. doi: 10.7554/eLife.99937 (PMC12582567; doi:10.7554/eLife.99937)
Supplement: Figure 5—figure supplement 2—source data 1. [file elife-99937-fig5-figsupp2-data1.pdf]

Source data 1. PDF file containing original western blots and EMSA for **Figure 5-figure supplement 2D and E**, showing the relevant bands boxed and labelled.

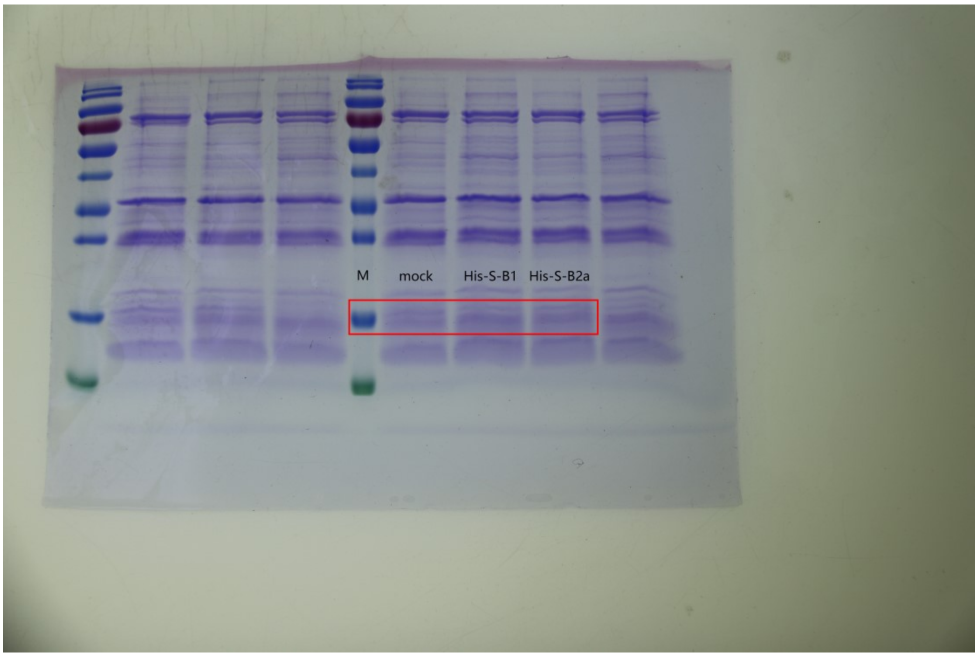

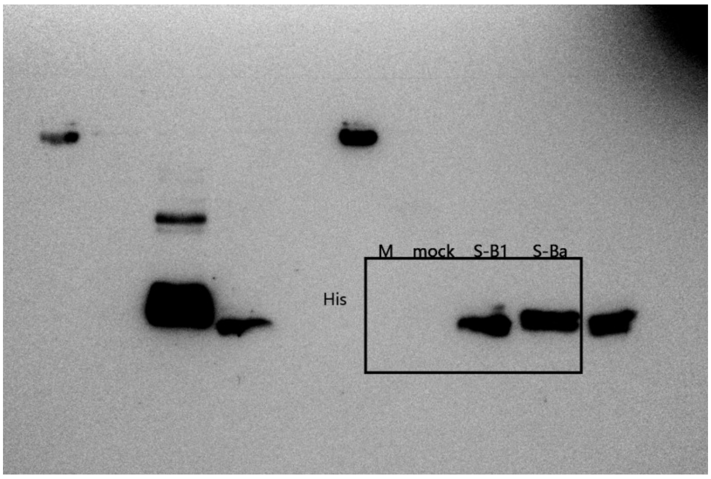

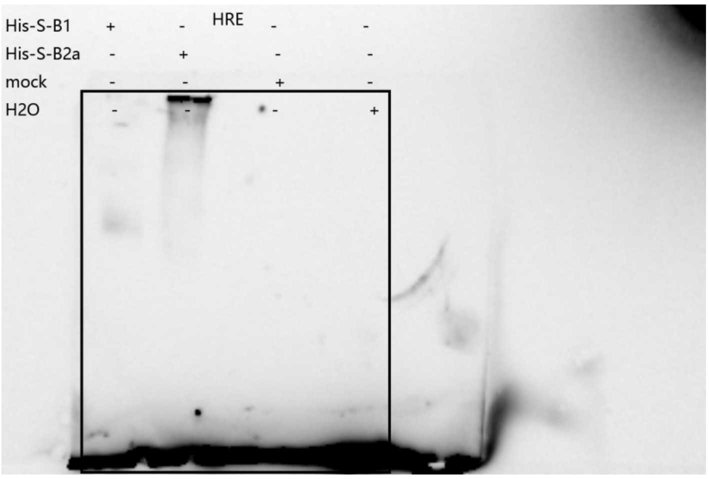
HRE
His-S-B1 + - - -
His-S-B2a - + - -
mock - - + -
H2O - - - +

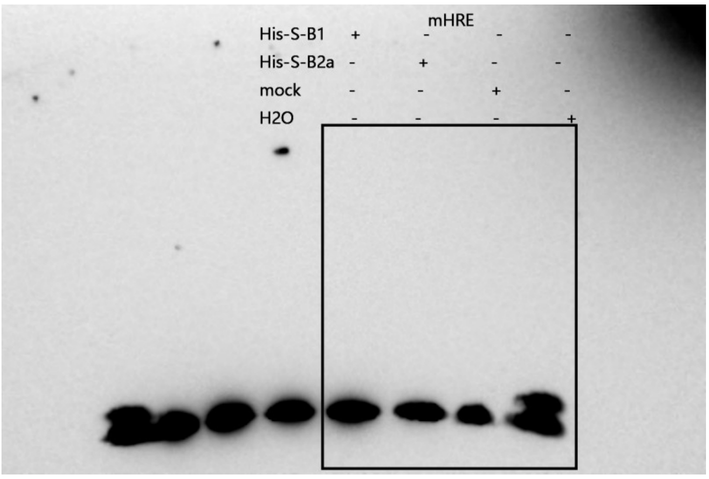
mHRE
His-S-B1 + - - -
His-S-B2a - + - -
mock - - + -
H2O - - - +

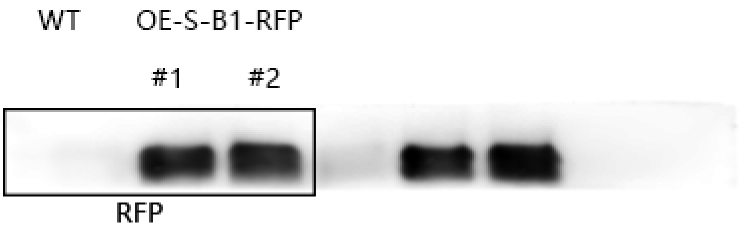
WT
OE-S-B1-RFP
#1
#2
RFP

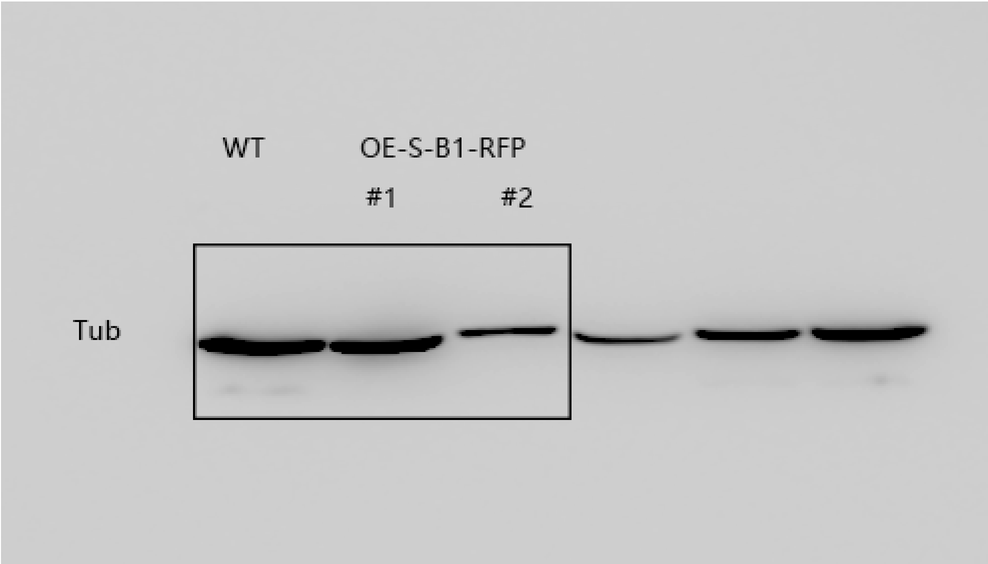
WT
OE-S-B1-RFP
#1
#2
Tub
