# Supplementary material for: Short heat shock factor A2 regulates heat resistance and growth balance in Arabidopsis
Source: eLife. 2025 Nov 3;13:RP99937. doi: 10.7554/eLife.99937 (PMC12582567; doi:10.7554/eLife.99937)

Source data 1. PDF file containing original western blot for **Figure 6–figure supplement 1A**, showing the relevant bands boxed and labelled.

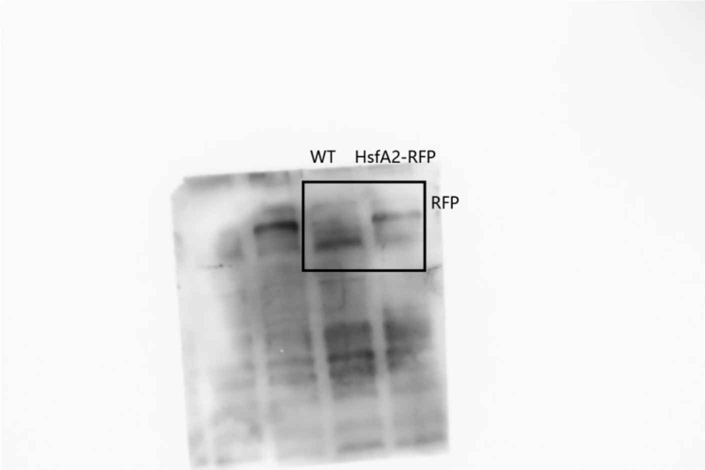

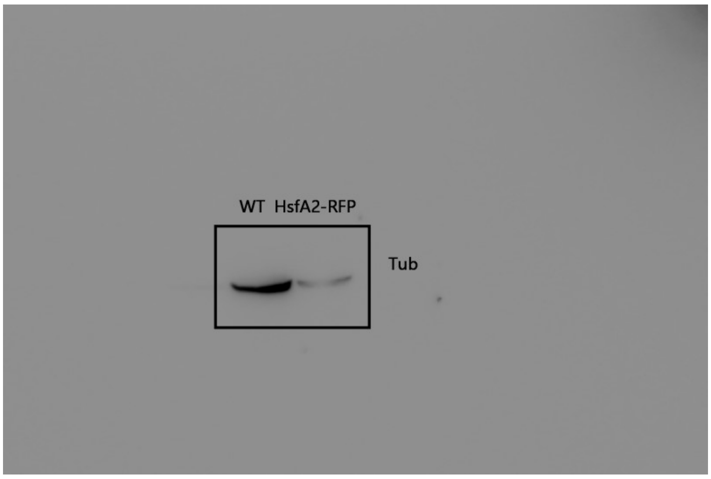

Supplement: Figure 6—figure supplement 1—source data 1. [file elife-99937-fig6-figsupp1-data1.pdf]
